# Type I IFN and not TNF, is Essential for Cyclic Di-nucleotide-elicited CTL by a Cytosolic Cross-presentation Pathway

**DOI:** 10.1016/j.ebiom.2017.07.016

**Published:** 2017-07-19

**Authors:** Darío Lirussi, Thomas Ebensen, Kai Schulze, Stephanie Trittel, Veronica Duran, Ines Liebich, Ulrich Kalinke, Carlos A. Guzmán

**Affiliations:** a Department of Vaccinology and Applied Microbiology, Helmholtz Centre for Infection Research, Braunschweig, Germany; b Institute for Experimental Infection Research, TWINCORE, Centre for Experimental and Clinical Infection Research, A Joint Venture Between the Helmholtz Centre for Infection Research and the Hannover Medical School, Hannover, Germany; c geneXplain GmbH, Wolfenbüttel, Germany

**Keywords:** Vaccine, CDN, CDA, CTL, Type I IFN, Cross-presentation, Cytosolic pathway

## Abstract

Cyclic di-nucleotides (CDN) are potent stimulators of innate and adaptive immune responses. Cyclic di-AMP (CDA) is a promising adjuvant that generates humoral and cellular immunity. The strong STING-dependent stimulation of type I IFN represents a key feature of CDA. However, recent studies suggested that this is dispensable for adjuvanticity. Here we demonstrate that stimulation of IFN-γ-secreting CD8^+^ cytotoxic T lymphocytes (CTL) is significantly decreased after vaccination in the absence of type I IFN signaling. The biological significance of this CTL response was confirmed by the stimulation of MHC class I-restricted protection against influenza virus challenge. We show here that type I IFN (and not TNF-α) is essential for CDA-mediated cross-presentation by a cathepsin independent, TAP and proteosome dependent cytosolic antigen processing pathway, which promotes effective cross-priming and further CTL induction. Our data clearly demonstrate that type I IFN signaling is critical for CDN-mediated cross-presentation.

## Highlights

• Cyclic di-AMP is a potent adjuvant with a proven capacity to promote cellular and humoral immunity.
• It was postulated that type I IFN is irrelevant for CDA-generated immunity.
• We show that CDA-mediated IFN-α/β signaling is essential for cytosolic cross-presentation, generating CTL by cross-priming.

Antigen cross-presentation generates cross-priming of CD8 cytotoxic T cells (CTL), which kill intracellular pathogens and cancer cells. The generation of CTL is the main feature of cyclic di- nucleotide adjuvants (CDN). The production of type I IFN has been shown to be essential for the generation of CTL by other adjuvants, but not for cyclic di-AMP, a paramount CDN. Surprisingly, it was recently claimed that type I IFN is dispensable for the immunogenicity of CDN. We show here that type I IFN (and not TNF) is necessary for the cytosolic cross-presentation pathway activated by CDN, which results in CTL generation. This finding and the knowledge of the mechanism are of crucial importance for the safe development of CDN-adjuvanted vaccines.

## 1 Introduction

Cyclic di-nucleotides (CDN) are conserved second messengers in prokaryotes, having important roles in metabolism, defense and cell-to-cell communication (Sureka et al., 2014). Thus, CDN are surveilled as faithful indicators of pathogen presence in higher eukaryotes, which allows their inclusion as members of pathogen-associated molecular patterns (PAMPs) (Dey et al., 2015). CDN are also generated endogenously in eukaryotes in the presence of foreign nucleic acids (Gao et al., 2013). CDN can stimulate innate and adaptive immune responses, thus representing promising vaccine adjuvants (Ebensen et al., 2007, Libanova et al., 2011). In contrast to most adjuvants licensed for human use or under development, CDN are able to promote both antibody and cytotoxic T lymphocyte (CTL) responses, being active by both systemic and mucosal routes (Ebensen et al., 2011, Wang and Celis, 2015). CDN interact directly with a stimulator of interferon genes (STING) (Sauer et al., 2011, Yin et al., 2012) and other cytosolic mediators, which results in a copious production of type I IFN (Konno et al., 2013, Parvatiyar et al., 2012). Type I IFN seems to be required for the induction of a proper immune response, particularly in the development of cellular immunity (Durbin et al., 2013, Liu et al., 2011, Levy et al., 2011). Thus, its CTL generation capabilities renders CDN top candidates not only for prophylactic vaccines, but also for immune interventions in the infection and cancer fields (Fu et al., 2015, Hanson et al., 2015, Demaria et al., 2015, Corrales et al., 2015), leading to ongoing clinical trials (Aduro Biotech and Pharmaceuticals, 2016). Nevertheless, the elucidation of the underlying mechanisms of action is a prerequisite for a safe implementation of CDN vaccines. Moreover, it has been recently postulated that type I IFN is dispensable for CDN-mediated adjuvanticity (Blaauboer et al., 2014, Hanson et al., 2015). Considering the body of experimental evidence suggesting that type I IFN can support stimulation of CTL responses (Le Bon et al., 2003, Le Bon and Tough, 2008, Schiavoni et al., 2013, Tovey et al., 2008), we hypothesized that CDN-mediated activation of type I IFN signaling (and not TNF) must be required for cross-presentation and subsequent CTL generation, a crucial feature for CDN-mediated immune protection.

Our studies demonstrate that CDA promotes a type I IFN-dependent cytosolic cross-presentation pathway that is proteosome and TAP dependent, which is essential for the stimulation of a long-lasting CTL-dependent protection against infection.

## 2 Materials and Methods

### 2.1 Animals

C57BL/6 and OT-I mice were bred at the Helmholtz Centre for Infection Research (HZI) or purchased from Harlan (Rossdorf, Germany), Sting Gt/Gt (goldenticket, C57BL/6J-*Tmem173*^*gt*^/J) and Tnfr1a/b −/− (B6.129S-*Tnfrsf1a*^*tm1lmx*^ *Tnfrsf1b*^*tm1lmx*^/J) (Peschon et al., 1998) were purchased from Jackson Laboratory (Bar Harbor, ME, USA), and Ifn-β −/− (Erlandsson et al., 1998) and *Ifnar1* −/− (*Ifnar1*^*tm1Agt*^, Muller et al., 1994) mice bred at the HZI. All animals were on the C57BL/6 background and were kept under specific pathogen-free conditions. All experiments were performed in compliance with the German animal protection law (TierSchG BGBl. I S 1105; 25.05.1998) and were approved by the Lower Saxony Committee on the Ethics of Animal Experiments as well as the responsible state office (Lower Saxony State Office of Consumer Protection and Food Safety), under permit numbers 33.11.42502-04-105/07 and 33.4-42502-04-13/1281.

### 2.2 Immunizations

Mice were immunized s.c. or i.n. in a prime and boost regime (two doses) with a two-week-window between doses. Each dose consisted of 20 μg OVA (or 50 μg OVA for long-lasting memory) (EndoGrade, Hyglos, Germany) and 7.5 μg of CDA (Biolog, Bremen, Germany) or vehicle (PBS, Hyglos and Ampuwa; Serumwerk, Bernburg, Germany). After a two to three–week-window, vaccinated mice were tested for T cell responses or underwent pathogen challenge. Animals used for the analysis of long-lasting CTL memory where maintained for 8.5 months and received an antigen recall (OVA 50 μg) before performing the *in vivo* CTL assays. Some of the immunized animals were euthanized and spleen and LN were removed for lymphocyte isolation. The cells were then processed for IFN-γ secretion, production of intracellular cytokines and killing assays.

### 2.3 BMDC Generation

Mice bone marrows were diluted to 1 × 10^6^ cells/ml in RPMI 1640 supplemented with L-Glu 2 mM (Hyglos GmbH, Germany), 10% FCS, penicillin, streptomycin, gentamycin 50 μg/ml (Gibco, USA), and were culture with the addition of 20 ng/ml GM-CSF (BD, USA), which was replenished every 48 h for 6 days.

### 2.4 Adoptive Cell Transfers for *In Vivo* Proliferation

CD8^+^ T cells were isolated from spleen and LN of OT-I mice (Hogquist et al., 1994) using Miltenyi columns and stained with 1 μM CFSE. Then, 2 × 10^6^ OT-I CD8^+^ T cells were injected into the tail vein of WT B6 and *Ifnar1* −/− mice. After 24 h, mice were immunized by s.c. route with OVA, OVA + CDA or vehicle (50 μg of OVA and 7.5 μg of CDA per mouse). After 3–4 days of T cell transfer, mice were euthanized and spleen and LN were isolated (Herve et al., 2013). Proliferation of OT-I CD8^+^ T cells was measured by CFSE diming by flow cytometry.

### 2.5 *In Vitro* Cross-presentation

BMDC were loaded with 5 μg/ml OVA ± 5 μg/ml of CDA for 24 h in the presence of cross-presentation inhibitors or vehicle. In the case of US6(20-146), cells were incubated for 48 h, and the inhibitor (see Reagents below) was added during the last 12 h of incubation. CD11c^+^ cells were stained with live-dead exclusion marker and fluorophore-conjugated antibody against CD11c. Then, MHC I–SIINFEKL expression was detected by flow cytometry using the specific antibody conjugated to PE (Biolegend #141603).

### 2.6 *In Vivo* CTL Assay

*In vivo* cell lysis by CTL was measured as percentage of specific lysis of cells loaded with either OVA or its immunodominant MHC class I-restricted peptide SIINFEKL. Splenocytes were separated in three groups and incubated with SIINFEKL 1 μM, OVA 1 mg/ml, and vehicle as a negative control for lysis. The cells were stained with 0.05 (low), 0.5 (medium) and 5 μM (high intensity) CSFE, respectively, and then mixed and injected i.v. at 3 × 10^7^ cells/mouse. The percentage of specific lysis was calculated based on ratios: percentage of non-pulsed CFSE_high_ cells/percentage of OVA_medium_ or SIINFEKL_low_ pulsed cells. The percentage of specific lysis was then calculated as a normalization to control animals by the following formula: [1 − (ratio from control non-primed recipient mice / ratio from primed recipient mice)] ∗ 100 (Wang et al., 2005, Wang and Golding, 2005).

### 2.7 Lymphocyte Isolation, Intracellular Cytokine Staining

Spleens and LN were homogenized by mechanical smashing on cell strainer. Red blood cells from spleens were destroyed by ACK buffer lysis. Lymphocytes were washed with PBS and counted for subsequent subpopulation isolation by positive selection on LS columns (Miltenyi, Germany) or *in vitro* re-stimulation. For intracellular cytokine staining, lymphocytes were incubated overnight in the presence of antigen (OVA, 20 μg/ml) or vehicle. During the last 6 h of antigenic stimulation, cells were treated with brefeldin (5 μg/ml) and monensin (6 μg/ml). Cells were stained with antibodies specific for surface receptors and live/dead cell marker for 30 min at 4 °C (see Reagents below). Cells were then washed in PBS, fixed in 1% paraformaldehyde, and permeabilized with 0.5% saponin, 0.5% bovine serum albumin (BSA) in PBS for 1 h at 4 °C. Intracellular staining with cytokine specific antibodies (see Reagents) was performed in permeabilization solution for 45 min at 4 °C.

### 2.8 Confocal Microscopy and Confocal IF Analysis

BMDC were cultured in sterile cover slides in 24- or 12-well plates overnight. BMDC were later treated with different combinations of fluorescently-marked DQ-OVA, OVA-FITC in the presence or absence of either inhibitors or CDA. Cells were then fixed with 4% paraformaldehyde in a 4% sucrose solution in PBS at room temperature. Subsequently, reactive aldehyde was quenched by amide (glycine), and cells were permeabilized with 0.1% Triton-× 100 in PBS for 5 min. Cells were then stained with primary antibodies against TAP1 (M-18, sc-11465, Santa Cruz), Rab5 (clone C8B1, Cell Signaling) calnexin (C4731, SIGMA) and LAMP-3 (clone NVG-2, anti-CD63 conjugated to APC, eBioscience) overnight at 4 °C. After washing, cells were incubated with secondary antibodies (goat anti-rat/mouse/rabbit conjugated to A488, A546 and donkey anti-goat conjugated to A633, or A546, Molecular probes) for 45 min at RT. Nuclei were stained with Hoechst 33258 (Molecular Probes), slides were mounted with Immumount (Sigma). Images were acquired by a Leica TCS SP5 Confocal Laser Scanning Microscope (Leica Microsystems, Wetzlar, Germany).

### 2.9 Data Processing

Flow cytometry data were acquired on a LSR Fortessa with FACSDiva software (BD Biosciences, USA) and were analyzed with FlowJo software (FlowJo, LLC, USA). Other data were analyzed with Microsoft Excel and GraphPad Prism 5 statistical software (GraphPad Software Inc., USA). All observed differences were tested for statistical significance at *p* ≤ 0.05 by unpaired Student's *t*-test using GraphPad Prism 5. Image processing was done in Adobe Photoshop (Sedgewick, 2010) (Adobe Systems Inc., USA).

### 2.10 Viral Challenge

Vaccinated animals and controls received 10^5^ PFU of the H1N1 PR8 influenza A/WSN/33 (WSN)-OVA(I), which expressed the MHC class I-restricted SIINFEKL epitope from OVA within the hemagglutinin (Topham et al., 2001) by i.n. route under anesthesia (isoflurane). Body weight and health parameters (*e.g.*, piloerection and motility) were monitored daily during the first 12–15 days post infection.

### 2.11 Reagents

Leupeptin, *Pseudomonas* exotoxin A, proteosomal inhibitors lactacystin and MG-132, monensin and brefeldin A were purchased from Sigma-Aldrich (Steinheim, Germany). Primaquine and *Z*-FL-COCHO (CSI) were obtained from Calbiochem (Darmstadt, Germany), bovine serum albumin was from Roth (Karlsruhe, Germany), saponin from Serva (Heidelberg, Germany) and butabindide oxalate was obtained from Tocris Bioscience (Bristol, UK). DQ-OVA, live-dead UV-blue staining and CFSE were obtained from Molecular Probes (Eugene, Oregon). Anti-mouse antibodies: CD3 (clone 500A2, V500 conjugated) and anti-IL-2 (clone JES6-5H4, APC-Cy7 conjugated) were obtained from BD Bioscience (USA); anti-CD4 (clone RM4-5, PE-Cy7 conjugated), anti-CD11c (clone N418, APC conjugated), anti-TNF-α (clone MPG-XT22, PerCP-eF710 conjugated), anti-CD107a (eBio1D4B, FITC conjugated), anti-NKp46 (29A1.4, eFluor660), anti-IL-4 (clone 11B11, APC conjugated), and anti-Thy1.1 (clone HIS51, Pe-Cy7 conjugated) were obtained from eBioscience Inc. (USA); and anti-CD8 (clone 53-6.7, BV650 conjugated), anti-CD11c (clone N418, PB conjugated), anti-IFN-γ (clone XMG1.2, BV711 and BV785 conjugated), LEAF™ Purified anti-mouse TNF-α, and mouse recombinant GM-CSF were obtained from Biolegend (USA).

### 2.12 *In Vitro* Proliferation

BMDC were loaded with 5 μg/ml OVA ± 5 μg/ml of CDA for 24 h in the presence of cross-priming inhibitors or vehicle. CD11c^+^ cells were stained with live-dead exclusion marker and fluorophore-conjugated antibody against CD11c. Then, CFSE-labeled OT-I CD8^+^ T cells were co-cultured with antigen-pulsed BMDC for 5 days, and their proliferation was then measured by assessing the reduction in CFSE fluorescence intensity by flow cytometry.

### 2.13 IFN-γ Secretion Assay

For CD8^+^ T cell IFN-γ secretion assay, procedures were performed according kit instructions (Miltenyi, Germany). Briefly, splenocytes from vaccinated animals were incubated with anti-CD8 antibody conjugated to anti-IFN-γ capture antibody at 4° for 5 min, then washed and incubated for 45 min at 37° with continuous rotation for cytokine secretion. Cells were labeled with a PE-conjugated anti-IFN-γ and CD3 and CD8 specific antibodies as well as vital staining for 25 min at 4°, then washed and acquired in flow cytometer.

### 2.14 Degranulation Assay

For the assessment of the NK cell degranulation capacity, splenocytes were co-incubated with YAC-1 target cells (E:T ratio 10:1) and an anti-CD107a antibody for 6 h at 37 °C and 5% CO_2_. The internalization of CD107a as well as the cytokine secretion was prevented by adding monensin and brefeldin A (5 μg/ml) to the co-culture after 1 h incubation. After an additional 5 h of incubation, the staining for surface markers as well as intracellular IFN-γ was performed.

### 2.15 TAP Assay

Performed according to Fischbach et al. (2015), briefly, human PBMC were isolated from buffy coats of healthy donors using a Ficoll (Biocoll, Biochrom AG) gradient. PBMC were seeded in 24-well plates in a density of 1 × 10^6^ cells/well and the corresponding wells were incubated for 24 h with CDA to a final concentration of 5 μg/ml. After incubation, cells were harvested with PBS/EDTA 1 mM and stained for flow cytometry with anti CD3 in PerCP (clone UCHT1, Biolegend), anti-CD14 in BV421 (clone MφP9, BD Biosciences), anti-CD19 in Brilliant Violet 510 (clone HIB19, Biolegend), anti-HLA-DR in APC-Cy7 (clone L243, Biolegend) and anti-CD11c in PeCy7 (clone 3.9, Biolegend). After surface staining, 2 × 10^5^ cells were semipermeabilized using 300 ng of streptolysin O (Abcam) at 4 °C for 15 min and then washed to remove residual streptolysin. Peptide translocation was determined in the presence of 10 mM ATP/ADP and 10 mM NST-F fluorescent peptide in PBS buffer supplemented with 10 mM of MgCl_2_ for 15 min at 37 °C. The reaction was stopped with 150 μl of PBS/EDTA 20 mM. Cells were analyzed by flow cytometry directly after the reaction was stopped.

### 2.16 Microarray Analysis

BMDC were re-stimulated for 30 min, 2, 6, 24 and 48 h with Ovalbumin (Hyglos, Germany) alone or co-administered with 10 μg/ml CDA, to generate activated cells. Then, total RNA was isolated from collected DCs using Trizol (Invitrogen) and RNeasy Mini cleanup kit (Qiagen, Germany). RNA was quantified using a Nanodrop 1000D spectrophotometer (ThermoScientific, USA) and quality tested with Agilent 2100 Bioanalyser (Agilent Technologies, USA). Microarray data were generated from Affymetrix microarrays. A minimum of triplicate samples were analyzed to reach statistical significance.

The CEL files were uploaded into the geneXplain platform (Koschmann et al., 2015) for the following analysis: microarray measurements were normalized using Robust Multi-array Averaging (RMA) (Irizarry et al., 2003) in the package oligo (Carvalho and Irizarry, 2010). Differentially expressed genes (DEGs) were identified using limma (Smyth, 2005) for altogether 30 conditions defined by time point, treatment and age. Each condition was compared with control measurements to calculate fold changes (logFC) and significance of expression differences (adjusted *p*-values). Heatmaps were created using geneXplain platform. The microarray data discussed in Supplementary Fig. 5 and Table 1 were stored in NCBI's public repository Gene Expression Omnibus (GEO) under accession number GSE101466.

## 3 Results

### 3.1 CDA-mediated Stimulation of Cellular Immunity Depends on Type I IFN

**Fig. 1:**
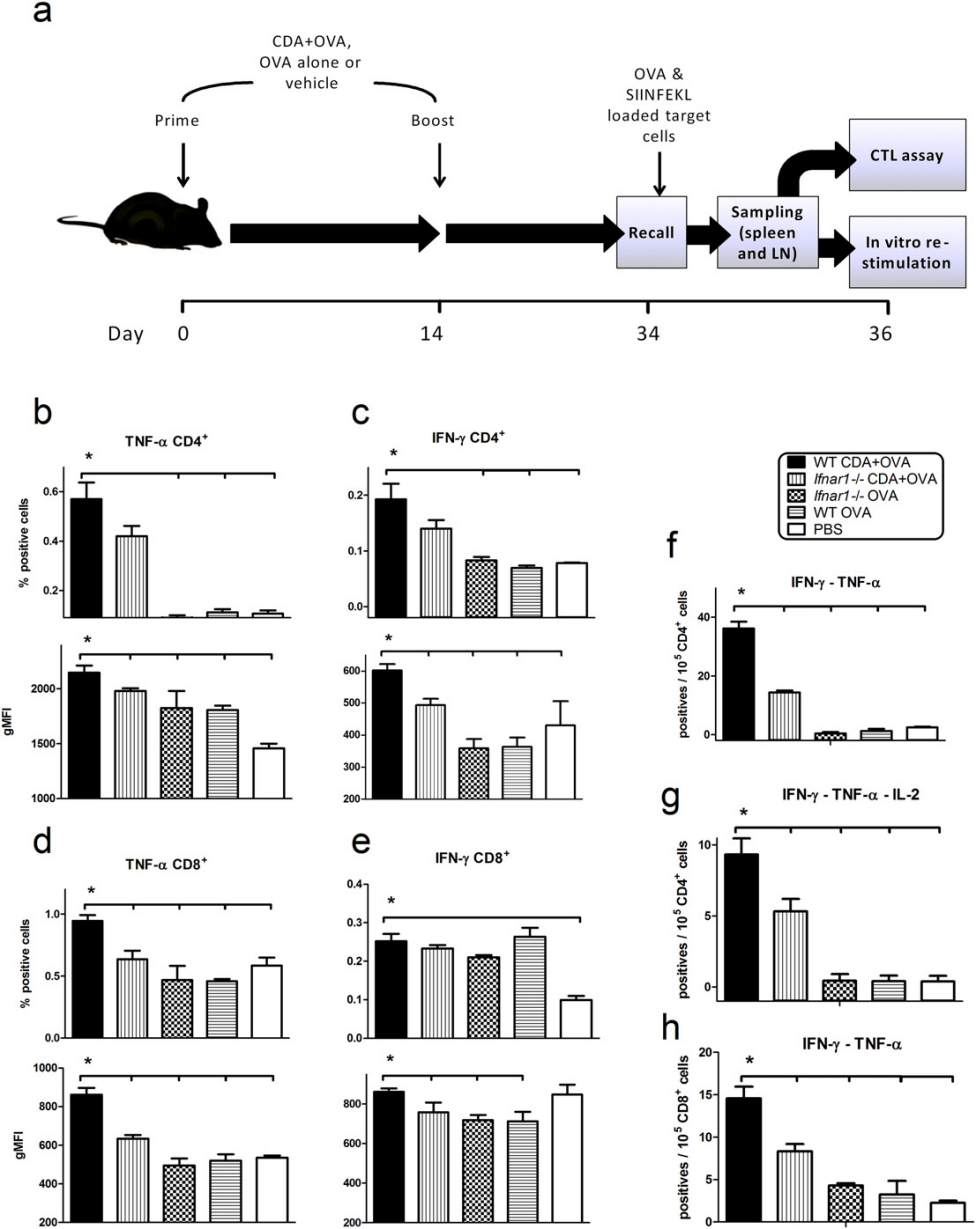
CDA-promoted cellular immunity depends on type I IFN signaling. a) C57BL/6 mice were immunized by intranasal (i.n.) route on day 0 and 14, and received a subsequent antigen challenge in form of primed target cells on day 34. On day 36 spleens and lymph nodes (LN) were isolated, and cell suspensions were prepared which were used for either determination of CTL activity or *in vitro* re-stimulation with soluble antigen to determine the production of intracellular cytokines by CD4^+^ and CD8^+^ T cell subsets by flow cytometry. b to e) Differential TNF-α (b and d) or IFN-γ (c and e) production from viable CD3^+^ CD4^+^ (b and c) or CD3^+^ CD8^+^ (d and e) T cells in the different treatment groups. Results are expressed as percentage of cells with relative intensities higher than 10^3^ (upper panels), and their geometric mean fluorescence intensity (gMFI, lower panels). f to h) Multifunctional CD4^+^ (f and g) CD8^+^ (h) T cells producing IFN-γ and TNF-α (f and h) or IFN-γ, TNF-α and IL-2 (g). Results are expressed as positive events per 10^5^ cells. The results are derived from a representative experiment out of three, with 4–5 animals per group, and vertical lines represent the SEM. The differences between WT CDA + OVA and the other treatment groups are statistically significant at *p* ≤ 0.05 (*) according to one tailed *t*-test.

Th1 responses are critical for the efficient generation of CTL responses, which are a landmark of the CDA-stimulated effector mechanisms activated following vaccination. Thus, we designed a vaccination strategy to simultaneously evaluate the role of type I IFN on CDA-dependent stimulation of Th and CTL responses (Fig. 1a). Wild type (WT) C57BL/6 mice and *Ifnar1* −/− mice, which lack type I IFN signaling, were immunized using CDA as adjuvant and ovalbumin (OVA) as a model antigen. Th1 responses were significantly decreased in *Ifnar1* −/− mice, as demonstrated by the reduction of both the number of cells expressing IFN-γ and TNF-α, and the amount of IFN-γ and TNF-α production (Fig. 1b,c and representative flow cytometry dot plots in Supplementary Fig. 1a). Furthermore, the number of multifunctional CD4^+^ T cells producing IFN-γ/TNF-α or IFN-γ/IL-2/TNF-α, as well as the stimulation of mono and multi-functional CD8^+^ T cells was significantly impaired in *Ifnar1* −/− mice (Fig. 1d–h and representative flow cytometry dot plots in Supplementary Fig. 1b). We found no differences in the frequency of CD8^+^ T cells between WT and *Ifnar1* −/− mice after vaccination or *in vitro* re-stimulation (Supplementary Fig. 1c). In addition, CTL responses have been described to be normal in *Ifnar1* −/− mice (Muller et al., 1994). The results obtained for IFN-γ positive CD8^+^ T cells were confirmed by a gold standard method for CTL detection, which consist of an IFN-γ specific secretion assay (Supplementary Fig. 1d and representative flow cytometry dot plots in Supplementary Fig. 1e).

### 3.2 Type I IFN is Essential for CDA-mediated CTL Responses

**Fig. 2:**
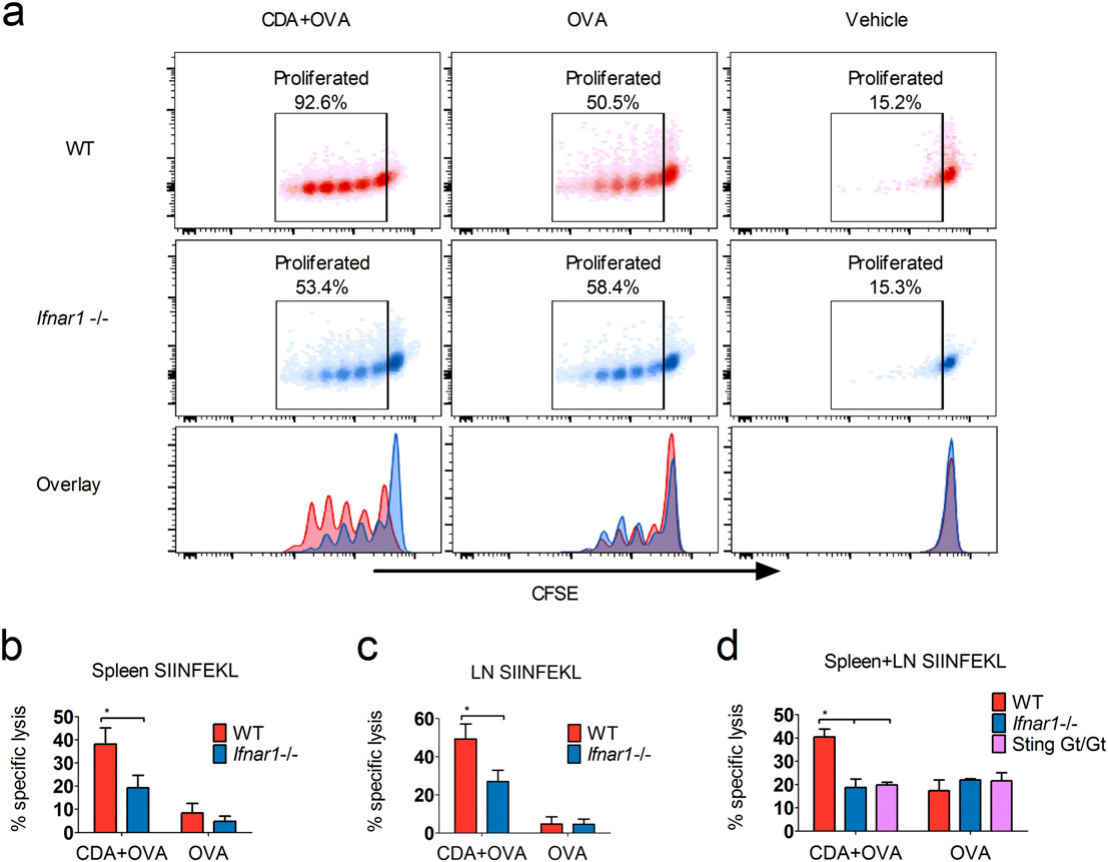
Type I IFN is essential for CDA-mediated stimulation of CTL responses. a) C57BL/6 WT and *Ifnar1* −/− mice received 2 × 10^6^ OT-I CD8^+^ T cells stained with 1 μM CFSE by i.v. route. CDA + OVA or OVA were injected by s.c. route 24 h later. The proliferation of the passively transferred OT-I CD8^+^ T cells was then measured by CFSE diming 3–4 days later of passive transfer. b to c) Induction of CTL in WT, Sting Gt/Gt and *Ifnar1* −/− mice vaccinated by i.n. (b and c) or s.c. (d) route according to scheme shown Fig. 1a. The percentages of lysis of SIINFEKL-loaded target cells were determined 16 h post i.v. injection in spleens (b), cervical lymph nodes (cLN) (c) or pooled samples (d). Percentages represent the specific lysis after subtraction of unspecific lysis in PBS controls (see Methods). Differences between the WT CDA + OVA and the other groups were statistically significant at *p* ≤ 0.05 (*) by one tailed *t*-test. Results show one representative out of 3 independent experiments with 3 (a) or 4–5 (b to d) animals per group, and vertical lines represent the SEM.

To further confirm the impact of type I IFN signaling on the generation of IFN-γ producing CD8^+^ T cells (mainly CTL), we assessed early activation of antigen-specific CD8^+^ T cells in vaccinated animals using a passive transfer model. To this end, we measured the proliferation of CFSE-stained OVA-specific CD8^+^ T cells derived from OT-I mice passively transferred into WT and *Ifnar1* −/− mice 24 h before administration of OVA alone (50 μg) or co-administered with CDA (7.5 μg). Proliferation of OT-I CD8^+^ T cells was greatly impaired in *Ifnar1* −/− mice as compared to WT mice (Fig. 2a). Next we evaluated the cytotoxic activity of the cells stimulated post vaccination by performing an *in vivo* CTL assay (Wang and Golding, 2005) in WT and *Ifnar1* −/− mice. The obtained results in CDA vaccinated animals demonstrated that the WT group were able to efficiently kill target cells in spleen and lymph nodes (LN), whereas *Ifnar1* −/− mice were significantly impaired on its CTL generation (Fig. 2b,c,d). These results were confirmed for the intra nasal (Fig. 2b, c) and sub cutaneous (s.c.) administration route (Fig. 2d), being thus independent of the vaccination route. Also in the s.c. administration SIINFEKL-loaded target cells were killed more efficiently in WT mice vaccinated with CDA + OVA as compared to those receiving OVA alone, whereas no differences were observed in *Ifnar1* −/− mice (Fig. 2d). The percentage of lysis in *Ifnar1* −/− mice was similar as in Goldenticket mice that lack Sting activity (Sting Gt/Gt). These animals were used as negative controls (Fig. 2d), since all effector activities of CDN are abolished in them (Sauer et al., 2011).

It has been reported that deficiencies in CTL activity in *Ifnar1* −/− mice can be affected by overreacting natural killer (NK) cells that deplete the CD8^+^ T cell subpopulation (Crouse et al., 2014, Xu et al., 2014). To rule out a potential contribution of this process to the observed phenotype, we assessed the biological activity of NK cells in mice receiving CDA as adjuvant. No significant differences were observed in the IFN-γ production or degranulation capacity of *Ifnar1* −/− NK cells compared to WT NK cells. However, NK cell responses were slightly diminished in *Ifnar1* −/− mice as compared with WT mice (Supplementary Fig. 2b,c). In conclusion, these results demonstrate that vaccinated *Ifnar1* −/− mice are defective in CTL responses, indicating the key role played by type I IFN in the stimulation of CTL when CDA is used as adjuvant.

### 3.3 CDN-mediated Cross-presentation of an Immunodominant CD8^+^ T Cell Epitope by DC Depends on Type I IFN

**Fig. 3:**
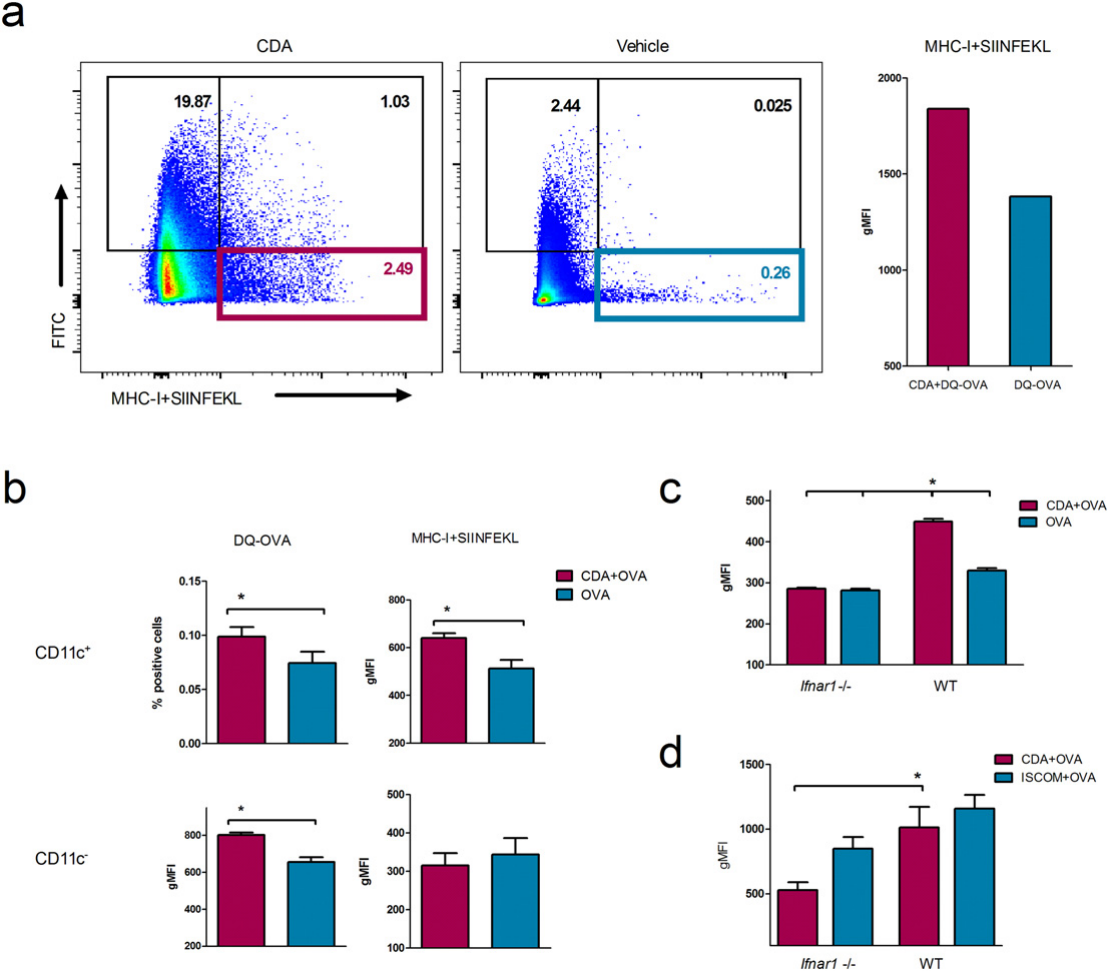
CDA-mediated cross-presentation of a CD8^+^ immunodominant epitope by CD11c^+^ cells is dependent on type I IFN. a) WT mice received DQ-OVA (500 μg) alone or co-administered with CDA (10 μg). After 4 h, animals were sacrificed and splenocytes were analyzed by FACS for antigen processing (DQ-OVA positive signal, FITC) and cross-presentation of the epitope (MHC-I–SIINFEKL PE positive signal). Percentages account for increased processing and cross-presentation after CDA treatment (left panels), whereas the gMFI of the PE signal (antibody 25-D1.16) shows an increase in cross-presentation (right panel). b) CDA induced processing and cross-presentation in CD11c^+^ and CD11c^−^ cells. BMDC from WT mice were incubated with DQ-OVA + CDA or DQ-OVA alone. Then, cells were analyzed for antigen processing (DQ-OVA^+^, left panels) and cross-presentation (MHC-I–SIINFEKL-PE^+^, right panels) by flow cytometry. c) MHC-I–SIINFEKL cross-presentation was analyzed by flow cytometry on CD11c^+^ gated BMDC from WT and *Ifnar1* −/− mice. d) CD11c^+^ BMDC were treated with CDA + OVA or ISCOMs + OVA 24 h. Differences were considered significant at *p* ≤ 0.05 (*) by one tailed *t*-test. Data correspond to a representative out of 2 (panel a) or 3 (panels b to d) experiments. Vertical lines represent SEM of triplicates.

Previous studies demonstrated that cross-presentation of a soluble antigen to CD8^+^ T cells can be facilitated by type I IFN (Le Bon et al., 2003). Thus, *in vitro* and *in vivo* experiments were performed to evaluate if type I IFN-supported cross-presentation is responsible for the CTL responses stimulated *in vivo* by CDA. In a first step, we evaluated cross-presentation *in vivo*. To this end, after intravenous (i.v.) injection of CDA + DQ-OVA or DQ-OVA alone, CD11c^+^ antigen presenting cells (APC) were analyzed for DQ-OVA processing (green fluorescent signal). Subsequently, cross-presentation of the immunodominant SIINFEKL CD8^+^ T cell epitope in the context of MHC-I molecules was assessed using the anti-MHC-I-SIINFEKL antibody 25-D1.16 (Porgador et al., 1997). Injection of CDA + DQ-OVA by i.v. route resulted in significantly increased levels of antigen processing by CD11c^+^ cells as compared to those observed after injection of DQ-OVA alone (Fig. 3a). Furthermore, the presentation of the processed SIINFEKL peptide in the context of MHC-I molecules was also clearly enhanced in animals receiving DQ-OVA co-administered with CDA (Fig. 3a). To rule out the contribution of other APC to the observed cross-presentation, we treated mouse splenocytes *in vitro* with DQ-OVA in the presence or absence of CDA. As expected, CD11c^+^ as well as CD11c^−^ cells (Fig. 3b, upper and lower panels, respectively) are capable of processing DQ-OVA. However, only CD11c^+^ cells were capable of cross-presenting SIINFEKL in the context of MHC-I molecules when CDA was present (Fig. 3b, right panels). Endotoxin free OVA cross-presentation levels were not significantly different from Medium background in CD11c^+^ gated splenocytes (Supplementary Fig. 3a) as well as in CD11c^+^ BMDC (data not shown).

Comparative studies were then performed using bone marrow derived dendritic cells (BMDC) from WT and *Ifnar1* −/− mice to assess if cross-presentation of the SIINFEKL epitope is impaired due to the lack of type I IFN signaling. CDA treatment resulted in significantly increased cross-presentation in cells derived from WT but not from *Ifnar1* −/− mice (Fig. 3c). In order to test if this type I IFN signaling dependent cross-presentation was shared among a different class of Sting ligands, we used DMXXA, as a potent mouse Sting agonist (Deng et al., 2014). We found that the cross-presentation generated by DMXXA was not significantly impaired in *Ifnar1* −/− BMDCs when compared to OVA controls (Supplementary Fig. 3b). The cross-presentation elicited by CDN was not affected in BMDC from mice deficient for TNF signaling (Supplementary Fig. 3c), thereby demonstrating that CDN-mediated activation of TNF-α (Blaauboer et al., 2014) is dispensable for cross-presentation. In order to assess the specificity of the CDA-induced cross-presentation, we used as control an adjuvant that does not elicit a major type I IFN production but promotes CTL responses (*i.e.*, ISCOMs) (Fossum et al., 2014). The cross-presentation levels were not significantly different in BMDC derived from WT and *Ifnar1* −/− mice treated with ISCOMs (Fig. 3e). This indicates that the effect of CDA in cross-presentation is dependent on stimulation of type I IFN signaling, and that this is not in turn an artifact resulting from a potentially impaired cross-presentation mechanism in *Ifnar1* −/− cells.

### 3.4 Characterization of the CDA-mediated Cross-presentation Pathway

**Fig. 4:**
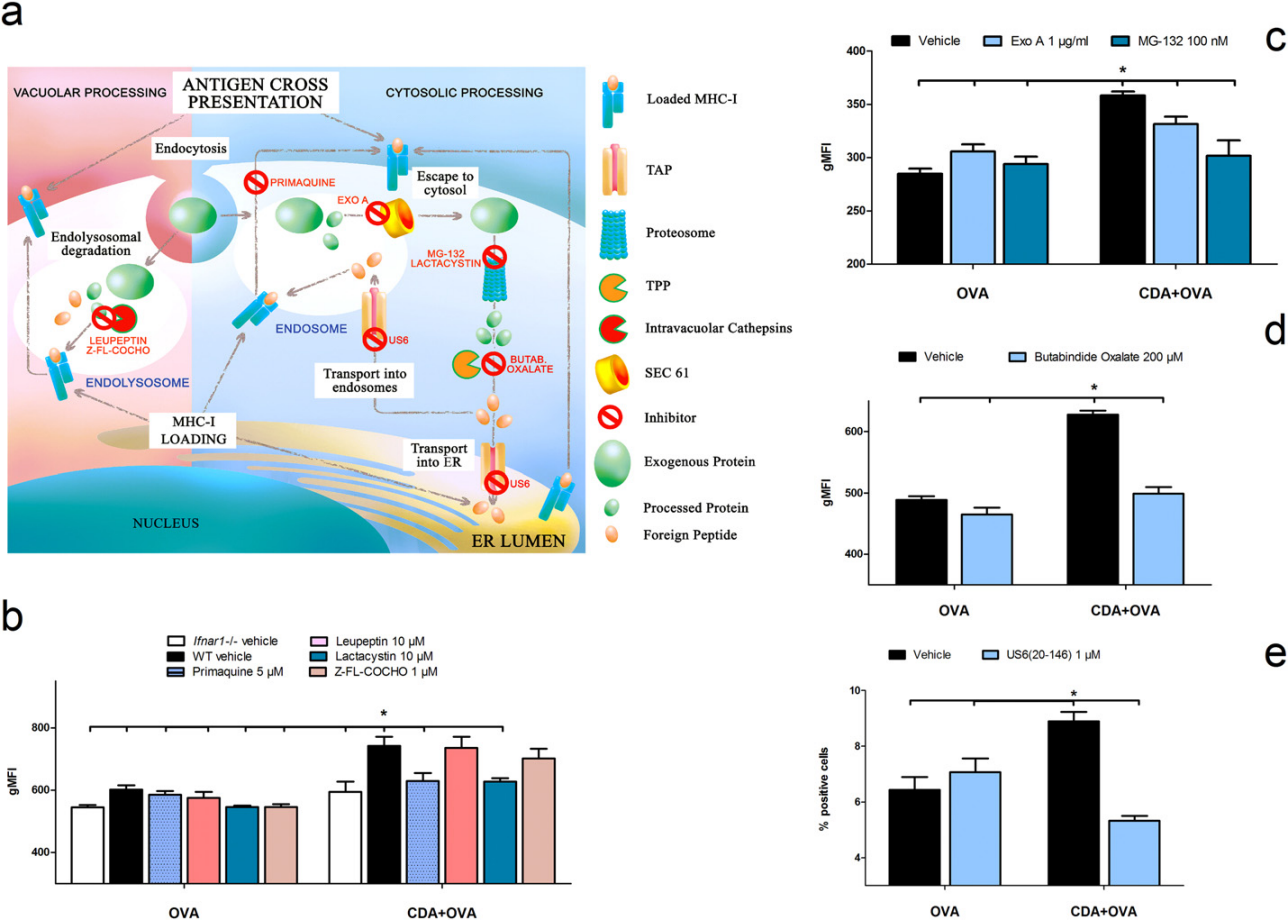
Dissection of the underlying mechanism of CDA-mediated cross-presentation. a) Vacuolar (left side) and cytosolic (right side) pathways for antigen cross-presentation. Inhibitors of key steps are shown in red. b to e) BMDC from WT mice were treated with CDA + OVA or OVA alone for 24 h (b to d) or 48 h (e) and cross-presentation of the SIINFEKL epitope was then detected by flow cytometry using the antibody 25-D1.16-PE. b) BMDC were treated in the absence or presence of inhibitors of the vacuolar pathway (leupeptin and *Z*-FL-COCHO), the cytosolic pathway (lactacystin) and of endosome secretion (primaquine). BMDC from *Ifnar1* −/− mice were used as negative control. c to e) BMDC were treated in the presence or absence of the inhibitors of components of the cytosolic pathway MG-132 (proteasome inhibitor) and Pseudomonas exotoxin A (ExoA, Sec61 translocon inhibitor) (c); butabindide oxalate (tripeptidyl-peptidase II inhibitor) (d); and US6 20-146aa (TAP inhibitor) (e). US6 20-146aa was added during the last 12 h of treatment, whereas the other inhibitors were present during the whole treatment. Results are expressed as gMFI (b to d) or percentage of positive cells (e) of at least triplicates in one representative out of two independent experiments, vertical lines represent SEM. Differences are statistically significant at *p* < 0.05 (*) by one tailed *t*-test. (For interpretation of the references to colour in this figure legend, the reader is referred to the web version of this article.)

Two main mechanisms for cross-presentation have been reported so far. The first, called the “vacuolar pathway”, involves intra-vacuolar cathepsines acting on antigen processing and the generation of peptides fitting into the MHC-I groove. The second, the so-called “cytosolic pathway”, involves antigen escape from endosomes to the cytosol, with subsequent degradation by the proteosome and subsidiary cytosolic proteases (Lin et al., 2008). It has also been shown for adjuvanted soluble antigen that cross-presentation in MHC-I is dependent on the export of endocytosed antigen from endosomes to the cytosol *via* the translocon protein Sec61, proteosomal processing into peptides in the cytosol, and importing back into the endosomes or endoplasmic reticulum (ER) for loading onto MHC-I complexes (Burgdorf et al., 2008) (Fig. 4a).

Despite the fact that type I IFN has been associated with the capacity to induce under certain circumstances cross-presentation, the underlying mechanism is unknown. Furthermore, even if a mechanism would be known, this would not be universally applicable to the mode of action of CDN. Thus, to assess which cross-presentation mechanism is important in CDA-mediated immunity, we first investigated the effects of selective inhibitors. The inhibitors of the “vacuolar pathway” like leupeptin (a calpain and cathepsin B inhibitor) (Momiyama et al., 2006) and *Z*-FL-COCHO (a cathepsin S inhibitor) did not affect cross-presentation of SIINFEKL by CDA + OVA or OVA-treated CD11c^+^ cells from WT mice. In contrast, the use of chemicals that affects the “cytosolic pathway”, such as lactacystin (a proteosomal inhibitor), significantly reduces CDA-mediated cross-presentation (Fig. 4b). In addition, we found that primaquine, which interferes with the trafficking of recycled endosomes to the cell membrane without affecting the classical endogenous MHC-I presentation at the level of ER or Golgi (van Weert et al., 2000), also inhibited CDA-dependent cross-presentation (Fig. 4b). The observation of the involvement of the “cytosolic pathway” is further strengthened by the significant inhibition of CDA-mediated cross-presentation detected using the alternative proteosome inhibitor MG-132 (Fig. 4c).

It was not clear until recently how antigens can escape first from the endosomal compartment in order to reach the proteosome. One of the confirmed mechanisms is the use of the translocon protein Sec61, whose inhibition abrogates cross-presentation (Zehner et al., 2015). In order to test if this is a part of the cross-presentation pathway enhanced by CDA, we used *Pseudomonas* exotoxin A, an inhibitor of Sec61 (Baleeiro et al., 2015, Zehner and Burgdorf, 2015). We found that inhibition of Sec61 significantly reduced CDA-triggered cross-presentation (Fig. 4c). To investigate the potential role of other cytosolic proteases on CDA-mediated cross-presentation, we used butabindide oxalate, an inhibitor of tripeptidyl-peptidase II, an enzyme that trims 15 aa long proteasome-derived peptides into smaller peptides suitable for MHC-I loading (Reits et al., 2004). Treatment with butabindide oxalate significantly inhibited cross-presentation (Fig. 4d). Cytosolic peptides have been reported to depend on TAP for cross-presentation (Brossart and Bevan, 1997, Ackerman et al., 2006, Song and Harding, 1996), since TAP can import peptides from cytosol not only into the ER but also into endosomes (Zehner et al., 2015, Burgdorf et al., 2008). We observed that inhibition of the TAP transport by a truncated US6 protein (US6 20-146aa) (Burgdorf et al., 2008) significantly decreased CDA-mediate cross-presentation (Fig. 4e), providing evidence on TAP transport contribution. To further validate these results, we performed a functional TAP assay (Fischbach et al., 2015). The obtained results confirmed the importance of TAP activity for CDA-mediated cross-presentation. TAP peptide transport was higher in the presence of CDA at different time points, reaching its maximum after 4 h of incubation (Supplementary Fig. 4). These experimental results were supported by transcriptional profiling of BMDC stimulated with CDA, OVA or CDA + OVA. The results showed that most of the genes involved in the vacuolar antigen processing pathway were not up-regulated over the first 48 h of treatment, containing the most downregulated genes. In contrast, the genes involved in the cytosolic antigen processing pathway (*e.g.*, Tap1, Tap2, Tapbp1 and Psmb8 and 9) were upregulated 24 h post CDA treatment, remaining upregulated for at least 48 h in the CDA + OVA group. Coincidentally, many of these cytosolic antigen processing genes were also type I IFN up-regulated genes (Supplementary Fig. 5 and Table 1).

We then confirmed the relevance of the observed differences on cross-presentation of the immunodominant SIINFEKL epitope on cross-priming of CD8^+^ T cells. BMDC from WT mice treated for 24 h with OVA or OVA + CDA with or without inhibitors were subsequently co-cultivated with CFSE-stained OT-I CD8^+^ T cells, and their proliferative capacity was then assessed. The activation of CD8^+^ T cells closely resembled the cross-presentation profiles of SIINFEKL, since the proteosomal inhibitors MG-132 and lactacystin, as well as Sec61 inhibitor Exo A significantly inhibited proliferation (Supplementary Fig. 6). In summary, these results provide strong experimental evidence supporting the fact that CDA stimulates cross-presentation *via* the “cytosolic pathway”.

### 3.5 Microscopic Analysis of Antigen Subcellular Fate in CDA-treated Cells

**Fig. 5:**
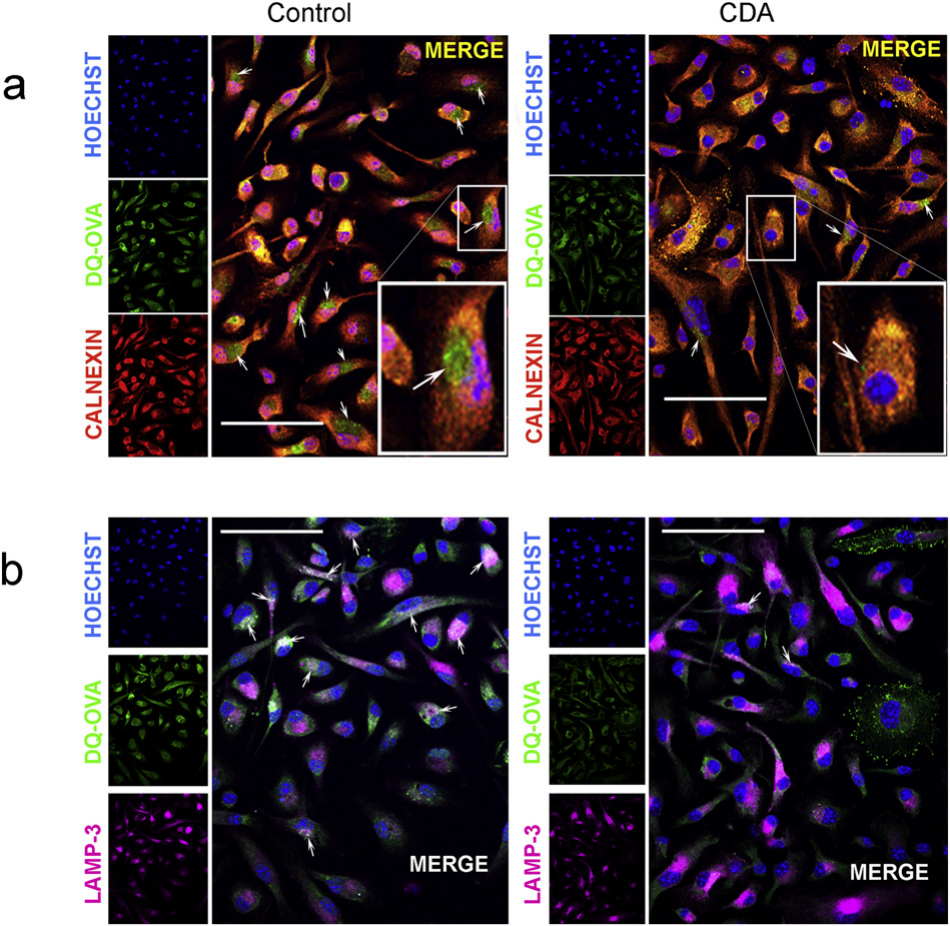
Microscopic analysis of antigen processing after CDA treatment. BMDC were treated with DQ-OVA (20 μg/ml) in the presence or absence of CDA (5 μg/ml) for 4 h (right and left panels respectively). a) Antigen processing was visualized in the green channel and ER marker calnexin was visualized in the red channel. In control cells the merged signals show large perinuclear speckles of processed OVA (green areas, arrows), which do not co-localize with the ER marker calnexin. In contrast, a high degree of co-localization is evident in CDA-treated cells, in which only a few tiny speckles of non-co-localizing OVA are observed (arrow). b) Cellular staining for the lysosomal marker LAMP-3 and co-localization with processed DQ-OVA. In the absence of CDA, a high number of co-localization spots are shown (arrows), which are considerably reduced in CDA-treated cells (right panel, arrows). Nuclear staining was performed with Hoechst 33258. Bars = 25 μm. (For interpretation of the references to colour in this figure legend, the reader is referred to the web version of this article.)

To further characterize the mechanism of action of CDA on antigen cross-presentation, the subcellular fate of fluorophore-labeled OVA or DQ-OVA was analyzed. Based on the results obtained with the inhibitors, we hypothesized that CDA drives an early antigen processing in the cytosol following escape from the endosomal compartment. For MHC-I loading, it would be expected that the processed peptides should return to vacuoles that contain key processing molecules from the ER, such as calnexin. Thus, we evaluated if CDA-treatment results in co-localization of the processed antigen and calnexin. Incubation of BMDC with DQ-OVA + CDA for 4 h resulted in stronger co-localization signals than cells treated with DQ-OVA alone. Furthermore, large subcellular accumulations of processed DQ-OVA were observed in the absence of CDA (arrows), whereas those spots were reduced in number and size when cells were incubated with CDA (Fig. 5a). The spatial distribution of the processed antigen was also significantly different depending on the treatment. While BMDC treated with OVA alone displayed the canonical ER perinuclear staining pattern, in CDA-treated cells the spots were scattered in the periphery of the cytoplasm (Supplementary Fig. 7a). Previous studies showed that after receptor-mediated endocytosis, OVA is incorporated into endosomes that are fused with lysosomes, thereby generating LAMP-3 positive vacuoles (Salik et al., 1999). However, in CDA-treated cells DQ-OVA co-localization with LAMP-3 was reduced as compared with cells receiving DQ-OVA alone, as demonstrated by the number and size of positive spots (Fig. 5b, arrows). By using OVA-FITC and antibodies against the early endosomal marker Rab5, we were able to detect increased antigen uptake in the endosomal fraction under CDA treatment after 10 to 20 min (data not shown). We further detected an increased cytosolic antigen signal and a reduction of antigen co-localization within the endosomal compartment (Rab5, red channel) 4 h after a 15 min antigen pulse in CDA-treated BMDC, as compared with cells treated with antigen alone (Supplementary Fig. 7b). This observation made after 4 h of antigen administration is in agreement with our hypothesis of cytosolic proteosomal degradation for further ER processing during CDA-induced OVA cross-presentation. Altogether, the early presence of cytosolic OVA, the increased co-localization within ER of the processed antigen and its diminished co-localization with the lysosomal marker further support the CDA-mediated cytosolic pathway of antigen processing and cross-presentation.

### 3.6 CDA Adjuvantation of Vaccines Mediates Long-lasting CTL Immune Memory

**Fig. 6:**
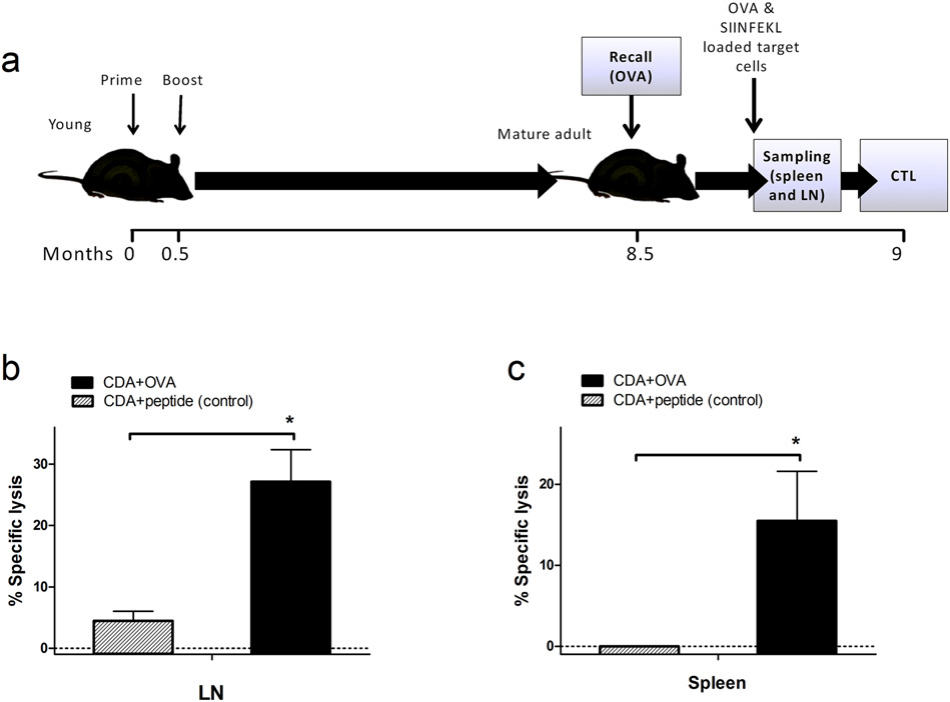
CDA-adjuvantation promotes long-lasting CTL immune memory. a) Vaccination scheme: 7-week-old C57BL/6 mice (young) were vaccinated by s.c. route on days 0 and 15 (50 μg of OVA alone or co-administered with 7.5 μg or CDA), after 8.5 months they received an antigen recall with OVA (50 μg), mice were sacrificed 14 days later and the *in vivo* stimulation of CTL responses was evaluated by flow cytometry. b and c) Specific lysis of SIINFEKL-pulsed target cells 40 h after transfer in LN (b) or spleens (c) of animals vaccinated with CDA + OVA or negative controls receiving CDA plus a MHC class II-restricted OVA peptide (OVA323–339). Differences were considered statistically significant at *p* < 0.05 (*) by one tailed *t*-test. Vertical lines represent SEM of 5 mice per group.

We next analyzed if long-term protective CTL responses can be obtained following vaccination using CDA as adjuvant. Mice primed and boosted with CDA-OVA on days 0 and 15 received a recall 9 months later (50 μg of OVA) and after 2 additional weeks CTL responses were evaluated (Fig. 6a). Animals vaccinated with CDA + OVA_323–339_ (MHC class II-restricted epitope, negative control) were not able to mount a CTL response against SIINFEKL-loaded cells in spleen and lymph nodes, even after an OVA recall. In contrast, a clear CTL response was observed when cells from spleen and lymph nodes from CDA + OVA-vaccinated animals were tested (Fig. 6b, c).

### 3.7 Role of CDA-mediated Type I IFN-dependent Stimulation of CTL in Protective Immunity

**Fig. 7:**
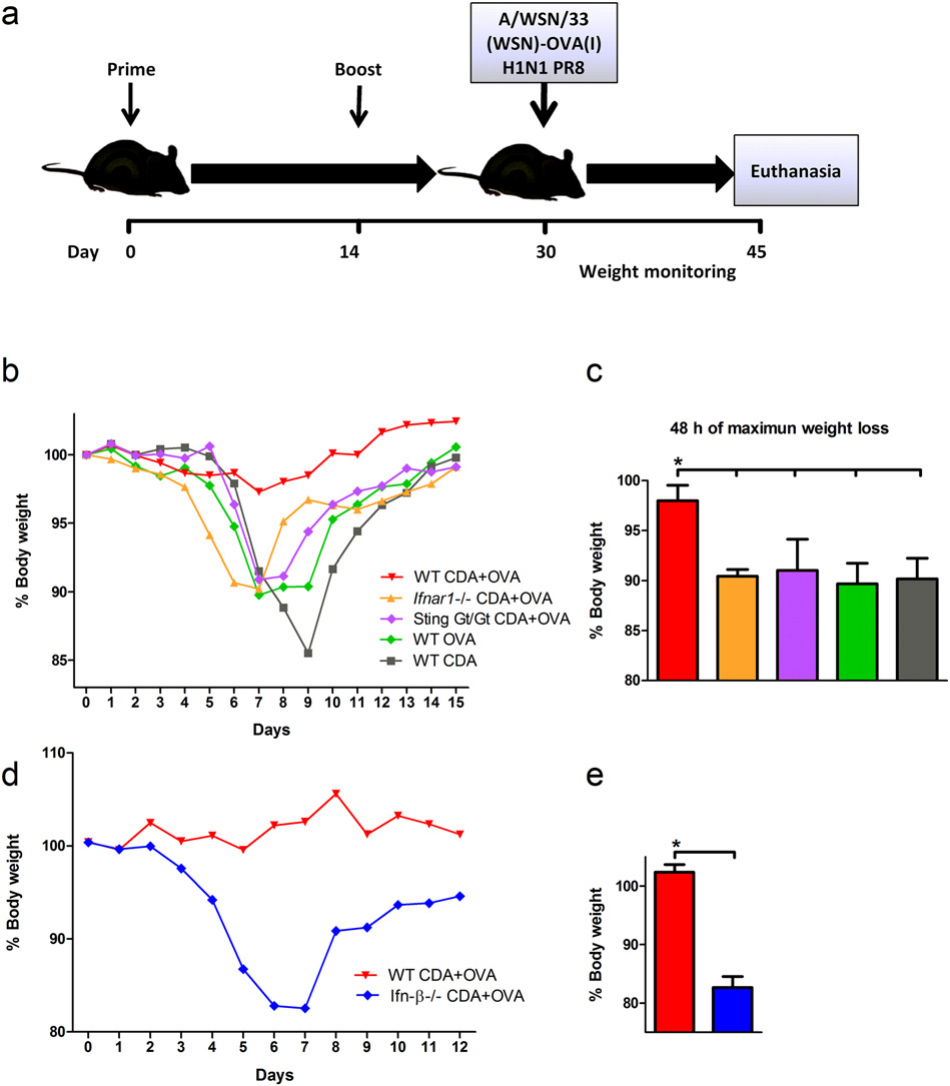
Role of CDA-mediated CTL responses in the stimulation of protective immunity post vaccination. WT, *Ifnar1* −/− and Sting Gt/Gt mice vaccinated with CDA + OVA, OVA alone or PBS were challenged by i.n. route with 10^5^ PFU of a recombinant H1N1 influenza PR8 strain expressing the SIINFEKL peptide of OVA integrated into hemagglutinin (A/WSN/33(WSN)-OVA I). a) Vaccination schedule and challenge timeline. b) Daily weight of 4–5 animals per group is plotted during two weeks post infection. c) Percentage of the initial body weight loss during the 48 h of maximum weight decrease. d) Viral challenge (10^5^ PFU) of vaccinated WT and Ifn-β −/− mice. Daily weight of animals (*n* = 4–5) during the first 12 days post infection. e) Percentage of the initial body weight loss during the 48 h of maximal weight change. Differences were considered statistically significant at *p* < 0.05 (*) using one tailed *t*-test. One representative out of 3 (a, b) or 2 (c, d) independent experiments is shown. Vertical lines represent the SEM.

We next assessed the impact of the type I IFN-mediated cross-presentation pathway in the protective immunity conferred by CDA-adjuvanted vaccines (Fig. 7a). To this end, mice vaccinated with CDA + OVA were challenged with a recombinant H1N1 influenza PR8 strain expressing the MHC class I-restricted SIINFEKL OVA peptide integrated into hemagglutinin (Topham et al., 2001). In this experimental infection model, OVA-mediated protection is dependent on the stimulation of SIINFEKL-specific CTL. As expected, mice lacking Sting signaling and control WT mice receiving OVA or CDA alone showed significant weight loss after challenge, as compared to WT mice vaccinated with CDA + OVA (Fig. 7b, c). On the other hand, *Ifnar1* −/− mice were also not protected, performing as bad as control mice and Sting Gt/Gt animals vaccinated with CDA + OVA (Fig. 7b, c). These data clearly indicate that type I IFN stimulation is essential for CDA-mediated protective CTL responses.

Additional studies were performed to assess the specific contribution of Ifn-β to both cross-presentation and the stimulation of protective immune responses. To this end, we performed a CTL assay in CDA immunized Ifn-β −/− mice. A reduction in the killing of SIINFEKL-pulsed cells was observed between Ifn-β −/− animals vaccinated with CDA + OVA and the WT counterparts (data not shown). To elucidate if the absence of Ifn-β also correlates with an impaired protection to infection in CDA-OVA-vaccinated animals, mice were challenged with the aforementioned SIINFEKL-expressing PR8 strain. The obtained results show that CDA + OVA-vaccinated WT mice were protected, whereas Ifn-β −/− mice immunized with CDA-OVA showed a significant weight loss on days 5 to 7 post infection (Fig. 7d, e). These data suggest that Ifn-β is crucial for CDA-mediated stimulation of CTL responses. However, we cannot completely rule out a potential contribution of IFN-α in the stimulation of CD8^+^ CTL.

## 4 Discussion

The generation of type I IFN was alternatively postulated as crucial (Dey et al., 2015), important (Abdul-Sater et al., 2013), and also dispensable (Blaauboer et al., 2014, Hanson et al., 2015) for CDN adjuvanticity. In this study we demonstrated that when CDA is used as adjuvant, CTL responses are only promoted in the presence of a functional type I IFN signaling, independently of the administration route. Since CD8^+^ T cells have been shown to be normally activated and to proliferate in an antigen specific mode in *Ifnar1* −/− mice (Dikopoulos et al., 2005), and the fact that we did not find any differences in the frequency of activated CD8^+^ T cells between *Ifnar1* −/− and WT mice (Supplementary Fig1c), we hypothesized that the cross-priming must be defective due to failed cross-presentation. We showed here that the proliferative capacity of passively transferred SIINFEKL-specific CD8^+^ OTI T cells and the induction of CTL were significantly diminished in *Ifnar1* −/− mice vaccinated with CDA + OVA, as compared to their WT counterparts. A similar decrease was observed in Sting deficient animals, in which CDN signaling is completely blocked. These *in vivo* CTL responses detected after vaccination indicated a CDA-dependent increment in cross-priming of SIINFEKL-specific CTL in WT mice, which was absent in Sting Gt/Gt or *Ifnar1* −/− mice.

Considering that cross-priming was impaired in *Ifnar1* −/− mice, we hypothesized that cross-presentation should also be impaired in their APC in the presence of CDA. The obtained results demonstrated that CDA increases antigen processing on different cell types, but it increases cross-presentation only in CD11c^+^ cells. This effect on CD11c^+^ cells is abrogated by the lack of type I IFN signaling, as shown when BMDC from *Ifnar1* −/− mice were tested. The fact that the lack of type I IFN signaling does not affect the cross-presentation promoted by other adjuvants such as ISCOMs, confirms both that cross-presentation is intact in *Ifnar1* −/− mice, and the importance of type I IFN signaling for CDA-mediated cross-presentation. In order to assess if the type-I IFN-dependent cross-presentation pathway induced by CDNs can be also promoted by other Sting ligands, *in vitro* experiments were performed using DMXAA. The cross-presentation elicited by DMXXA was independent on type I IFN signaling, demonstrating the existence of different underlying mechanisms triggered by Sting ligands. Moreover, we confirmed that CDN-mediated cross-presentation is independent of TNF-α, since CDN significantly increased cross-presentation in Tnfr1a/b −/− BMDC. Based on pre-existing knowledge on the effector functions of type I IFN, we postulated that the cross-presentation mechanism elicited by CDA must involve a canonical cytosolic pathway for antigen processing. Our experiments with inhibitors showed that CDA-mediated cross-presentation is indeed dependent on proteasome- and TPP-dependent antigen processing. Furthermore, we observed dependency on Sec61, TAP and endosome-to-cell membrane transport. The results showed that not only cross-presentation, but also cross-priming of CD8^+^ T cells is affected *in vitro* by inhibitors of the cytosolic pathway of cross-presentation. Nevertheless, to the best of our knowledge the dissection of type I IFN elicited cross-presentation pathway has not been reported before. Thus the results presented here indicates that the cross-presentation pathway favored by type I IFN comprises the export of antigen from endosomes to the cytosol by translocon Sec 61 (Zehner et al., 2015), cytosolic degradation by proteosome and TPP, and TAP-mediated transport of the generated peptides into an ER like compartment for MHC-I loading. We furtherly confirmed the importance of this pathway by microarray analysis.

All together, these data indicate that when type I IFN signaling is disabled there is a failure in the activation of CD8^+^ T cells by the cross-presentation mechanism triggered by CDA.

As for other described cross-presentation events depending on type I IFN, we can speculate that in the development of this mechanism, APC are able to generate an immune response against soluble antigens from invading pathogens. This will allow mounting a proper cellular killing arm of defense for non-canonically MHC-I-expressed epitopes. This mechanism itself seems to be evolved from cellular components primarily responsible for different cellular tasks. For example, the Sec61 translocon does not regularly export antigens from endosomes to cytosol for cross-presentation, but rather for the ER-associated degradation machinery (Fossum et al., 2014). The proteasome itself is naturally involved in MHC-I presentation of cytosolic ubiquitinated proteins, whereas the TAP transporter in conjunction with proteasomal processing allow the loading of ER resident MHC-I complexes (Burgdorf et al., 2008). Due to the need of a prompt response after pathogen pattern alert, this machinery can be used near the cell surface to rapidly load epitopes from endocytosed foreign protein on endosome resident MHC-I complexes. This spatial separation could speed the cross-presentation process in a manner that can change the outcome of the battle against an arising infection. Additional studies showed that animals immunized with CDA + OVA were able to mount a potent cellular response against the immunodominant CD8^+^ restricted SIINFEKL epitope shortly after an immunological recall with OVA nine months after the last immunization. Thus, the use of CDA as adjuvant promotes cellular immunological memory during long periods of time, a critical feature in terms of prophylactic vaccination.

To assess the biological significance of the type I IFN-dependent CTL responses stimulated by CDA, vaccinated WT and KO animals were challenged with a recombinant influenza virus carrying a CD8^+^ immunodominant epitope of OVA. The obtained results demonstrated that an intact type I IFN signaling pathway was crucial for achieving protection against viral challenge. In contrast to WT animals, *Ifnar1* −/− mice were not protected against infection post vaccination with CDA + OVA. Additional studies suggested that IFN-β is a key mediator for this response, since IFN-β −/− mice were susceptible to viral challenge to a similar extent as *Ifnar1* −/− and Sting Gt/Gt mice.

In conclusion, our studies demonstrated that CTL activation by CDA is strictly dependent on the stimulation of type I IFN-mediated cross-presentation. Type I IFN induction by CDA is also required for optimal stimulation of Th1 responses and induction of multifunctional CD4^+^ and CD8^+^ T cells. These findings clarify a key aspect of CDN mode of action, demonstrating that the current assumption that type I IFN induction is dispensable for CDN adjuvanticity should be revised. As demonstrated here, type I IFN is indeed crucial for the most striking capacities of CDN, namely, the stimulation of a CTL response, and the modulation of activity of Th1 cells. This information is crucial for the implementation of CDN-based immune interventions in the clinic, as well as for the generation of CDN derivatives with improved biological properties.

## Funding Sources

This work was in part supported by grants from the EU (UniVax, contract No. 601738), the grant 0315890 from the BMBF in the context of the program Gerontosys 2 (GerontoShield) and the Helmholtz Association (HAI-IDR). These funding sources had no role in research design, data collection or its analysis, crafting of the manuscript or decision to submit results for publication.

## Conflict of Interest Statement

C.A.G. and T.E. are named as inventors in patents covering the use of CDA as adjuvant (PCT/EP 2006010693, EP/04.04.02/EPA 02007640, and PCT/EP2006011182). All other authors have declared that no conflict of interest exists.

## Appendix A Supplementary data

Supplementary material Image 1

## References

[bb0005] Abdul-Sater A.A., Tattoli I., Jin L., Grajkowski A., Levi A., Koller B.H., Allen I.C., Beaucage S.L., Fitzgerald K.A., Ting J.P., Cambier J.C., Girardin S.E., Schindler C. (2013). Cyclic-di-GMP and cyclic-di-AMP activate the NLRP3 inflammasome. EMBO Rep..

[bb0010] Ackerman A.L., Giodini A., Cresswell P. (2006). A role for the endoplasmic reticulum protein retrotranslocation machinery during crosspresentation by dendritic cells. Immunity.

[bb0015] Aduro Biotech, I. & Pharmaceuticals, N (2016). Study of the Safety and Efficacy of MIW815 (ADU-S100) in Patients With Advanced/Metastatic Solid Tumors or Lymphomas. https://ClinicalTrials.gov/show/NCT02675439.

[bb0020] Baleeiro R.B., Rietscher R., Diedrich A., Czaplewska J.A., Lehr C.M., Scherliess R., Hanefeld A., Gottschaldt M., Walden P. (2015). Spatial separation of the processing and MHC class I loading compartments for cross-presentation of the tumor-associated antigen HER2/neu by human dendritic cells. Oncoimmunology.

[bb0025] Blaauboer S.M., Gabrielle V.D., Jin L. (2014). MPYS/STING-mediated TNF-alpha, not type I IFN, is essential for the mucosal adjuvant activity of (3′-5′)-cyclic-di-guanosine-monophosphate in vivo. J. Immunol..

[bb0030] Brossart P., Bevan M.J. (1997). Presentation of exogenous protein antigens on major histocompatibility complex class I molecules by dendritic cells: pathway of presentation and regulation by cytokines. Blood.

[bb0035] Burgdorf S., Scholz C., Kautz A., Tampe R., Kurts C. (2008). Spatial and mechanistic separation of cross-presentation and endogenous antigen presentation. Nat. Immunol..

[bb0040] Carvalho B.S., Irizarry R.A. (2010). A framework for oligonucleotide microarray preprocessing. Bioinformatics.

[bb0045] Corrales L., Glickman L.H., McWhirter S.M., Kanne D.B., Sivick K.E., Katibah G.E., Woo S.R., Lemmens E., Banda T., Leong J.J., Metchette K., Dubensky T.W., Gajewski T.F. (2015). Direct activation of STING in the tumor microenvironment leads to potent and systemic tumor regression and immunity. Cell Rep..

[bb0050] Crouse J., Bedenikovic G., Wiesel M., Ibberson M., Xenarios I., Von Laer D., Kalinke U., Vivier E., Jonjic S., Oxenius A. (2014). Type I interferons protect T cells against NK cell attack mediated by the activating receptor NCR1. Immunity.

[bb0055] Demaria O., De Gassart A., Coso S., Gestermann N., Di Domizio J., Flatz L., Gaide O., Michielin O., Hwu P., Petrova T.V., Martinon F., Modlin R.L., Speiser D.E., Gilliet M. (2015). STING activation of tumor endothelial cells initiates spontaneous and therapeutic antitumor immunity. Proc. Natl. Acad. Sci. U. S. A..

[bb0060] Deng L., Liang H., Xu M., Yang X., Burnette B., Arina A., Li X.D., Mauceri H., Beckett M., Darga T., Huang X., Gajewski T.F., Chen Z.J., Fu Y.X., Weichselbaum R.R. (2014). STING-dependent cytosolic DNA sensing promotes radiation-induced type I interferon-dependent antitumor immunity in immunogenic tumors. Immunity.

[bb0065] Dey B., Dey R.J., Cheung L.S., Pokkali S., Guo H., Lee J.H., Bishai W.R. (2015). A bacterial cyclic dinucleotide activates the cytosolic surveillance pathway and mediates innate resistance to tuberculosis. Nat. Med..

[bb0070] Dikopoulos N., Bertoletti A., Kroger A., Hauser H., Schirmbeck R., Reimann J. (2005). Type I IFN negatively regulates CD8 + T cell responses through IL-10-producing CD4 + T regulatory 1 cells. J. Immunol..

[bb0075] Durbin R.K., Kotenko S.V., Durbin J.E. (2013). Interferon induction and function at the mucosal surface. Immunol. Rev..

[bb0080] Ebensen T., Schulze K., Riese P., Link C., Morr M., Guzman C.A. (2007). The bacterial second messenger cyclic diGMP exhibits potent adjuvant properties. Vaccine.

[bb0085] Ebensen T., Libanova R., Schulze K., Yevsa T., Morr M., Guzman C.A. (2011). Bis-(3′,5′)-cyclic dimeric adenosine monophosphate: strong Th1/Th2/Th17 promoting mucosal adjuvant. Vaccine.

[bb0090] Erlandsson L., Blumenthal R., Eloranta M.L., Engel H., Alm G., Weiss S., Leanderson T. (1998). Interferon-beta is required for interferon-alpha production in mouse fibroblasts. Curr. Biol..

[bb0095] Fischbach H., Doring M., Nikles D., Lehnert E., Baldauf C., Kalinke U., Tampe R. (2015). Ultrasensitive quantification of TAP-dependent antigen compartmentalization in scarce primary immune cell subsets. Nat. Commun..

[bb0100] Fossum C., Hjertner B., Ahlberg V., Charerntantanakul W., McIntosh K., Fuxler L., Balagunaseelan N., Wallgren P., Lovgren Bengtsson K. (2014). Early inflammatory response to the saponin adjuvant Matrix-M in the pig. Vet. Immunol. Immunopathol..

[bb0105] Fu J., Kanne D.B., Leong M., Glickman L.H., McWhirter S.M., Lemmens E., Mechette K., Leong J.J., Lauer P., Liu W., Sivick K.E., Zeng Q., Soares K.C., Zheng L., Portnoy D.A., Woodward J.J., Pardoll D.M., Dubensky T.W., Kim Y. (2015). STING agonist formulated cancer vaccines can cure established tumors resistant to PD-1 blockade. Sci. Transl. Med..

[bb0110] Gao P., Ascano M., Wu Y., Barchet W., Gaffney B.L., Zillinger T., Serganov A.A., Liu Y., Jones R.A., Hartmann G., Tuschl T., Patel D.J. (2013). Cyclic [G(2′,5′)pA(3′,5′)p] is the metazoan second messenger produced by DNA-activated cyclic GMP-AMP synthase. Cell.

[bb0115] Hanson M.C., Crespo M.P., Abraham W., Moynihan K.D., Szeto G.L., Chen S.H., Melo M.B., Mueller S., Irvine D.J. (2015). Nanoparticulate STING agonists are potent lymph node-targeted vaccine adjuvants. J. Clin. Invest..

[bb0120] Herve J., Dubreil L., Tardif V., Terme M., Pogu S., Anegon I., Rozec B., Gauthier C., Bach J.M., Blancou P. (2013). beta2-Adrenoreceptor agonist inhibits antigen cross-presentation by dendritic cells. J. Immunol..

[bb0125] Hogquist K.A., Jameson S.C., Heath W.R., Howard J.L., Bevan M.J., Carbone F.R. (1994). T cell receptor antagonist peptides induce positive selection. Cell.

[bb0130] Irizarry R.A., Hobbs B., Collin F., Beazer-Barclay Y.D., Antonellis K.J., Scherf U., Speed T.P. (2003). Exploration, normalization, and summaries of high density oligonucleotide array probe level data. Biostatistics.

[bb0135] Konno H., Konno K., Barber G.N. (2013). Cyclic dinucleotides trigger ULK1 (ATG1) phosphorylation of STING to prevent sustained innate immune signaling. Cell.

[bb0140] Koschmann J., Bhar A., Stegmaier P., Kel A., Wingender E. (2015). “Upstream analysis”: an integrated promoter-pathway analysis approach to causal interpretation of microarray data. Microarrays.

[bb0145] Le Bon A., Tough D.F. (2008). Type I interferon as a stimulus for cross-priming. Cytokine Growth Factor Rev..

[bb0150] Le Bon A., Etchart N., Rossmann C., Ashton M., Hou S., Gewert D., Borrow P., Tough D.F. (2003). Cross-priming of CD8 + T cells stimulated by virus-induced type I interferon. Nat. Immunol..

[bb0155] Levy D.E., Marie I.J., Durbin J.E. (2011). Induction and function of type I and III interferon in response to viral infection. Curr. Opin. Virol..

[bb0160] Libanova R., Becker P.D., Guzman C.A. (2011). Cyclic di-nucleotides: new era for small molecules as adjuvants. Microb. Biotechnol..

[bb0165] Lin M.L., Zhan Y., Villadangos J.A., Lew A.M. (2008). The cell biology of cross-presentation and the role of dendritic cell subsets. Immunol. Cell Biol..

[bb0170] Liu S.Y., Sanchez D.J., Cheng G. (2011). New developments in the induction and antiviral effectors of type I interferon. Curr. Opin. Immunol..

[bb0175] Momiyama J., Hashimoto T., Matsubara A., Futai K., Namba A., Shinkawa H. (2006). Leupeptin, a calpain inhibitor, protects inner ear hair cells from aminoglycoside ototoxicity. Tohoku J. Exp. Med..

[bb0180] Muller U., Steinhoff U., Reis L.F., Hemmi S., Pavlovic J., Zinkernagel R.M., Aguet M. (1994). Functional role of type I and type II interferons in antiviral defense. Science.

[bb0185] Parvatiyar K., Zhang Z., Teles R.M., Ouyang S., Jiang Y., Iyer S.S., Zaver S.A., Schenk M., Zeng S., Zhong W., Liu Z.J., Modlin R.L., Liu Y.J., Cheng G. (2012). The helicase DDX41 recognizes the bacterial secondary messengers cyclic di-GMP and cyclic di-AMP to activate a type I interferon immune response. Nat. Immunol..

[bb0190] Peschon J.J., Torrance D.S., Stocking K.L., Glaccum M.B., Otten C., Willis C.R., Charrier K., Morrissey P.J., Ware C.B., Mohler K.M. (1998). TNF receptor-deficient mice reveal divergent roles for p55 and p75 in several models of inflammation. J. Immunol..

[bb0195] Porgador A., Yewdell J.W., Deng Y., Bennink J.R., Germain R.N. (1997). Localization, quantitation, and in situ detection of specific peptide-MHC class I complexes using a monoclonal antibody. Immunity.

[bb0200] Reits E., Neijssen J., Herberts C., Benckhuijsen W., Janssen L., Drijfhout J.W., Neefjes J. (2004). A major role for TPPII in trimming proteasomal degradation products for MHC class I antigen presentation. Immunity.

[bb0205] Salik E., Tyorkin M., Mohan S., George I., Becker K., Oei E., Kalb T., Sperber K. (1999). Antigen trafficking and accessory cell function in respiratory epithelial cells. Am. J. Respir. Cell Mol. Biol..

[bb0210] Sauer J.D., Sotelo-Troha K., Von Moltke J., Monroe K.M., Rae C.S., Brubaker S.W., Hyodo M., Hayakawa Y., Woodward J.J., Portnoy D.A., Vance R.E. (2011). The N-ethyl-N-nitrosourea-induced Goldenticket mouse mutant reveals an essential function of Sting in the in vivo interferon response to Listeria monocytogenes and cyclic dinucleotides. Infect. Immun..

[bb0215] Schiavoni G., Mattei F., Gabriele L. (2013). Type I interferons as stimulators of DC-mediated cross-priming: impact on anti-tumor response. Front. Immunol..

[bb0220] Sedgewick J. (2010). Scientific Imaging with Photoshop: Methods, Measurement, and Output.

[bb0225] Smyth G., Gentleman R., Carey V., Dudoit S., Irizarry R., Huber W. (2005). Limma: linear models for microarray data. Bioinformatics and Computational Biology Solutions Using R and Bioconductor.

[bb0230] Song R., Harding C.V. (1996). Roles of proteasomes, transporter for antigen presentation (TAP), and beta 2-microglobulin in the processing of bacterial or particulate antigens via an alternate class I MHC processing pathway. J. Immunol..

[bb0235] Sureka K., Choi P.H., Precit M., Delince M., Pensinger D.A., Huynh T.N., Jurado A.R., Goo Y.A., Sadilek M., Iavarone A.T., Sauer J.D., Tong L., Woodward J.J. (2014). The cyclic dinucleotide c-di-AMP is an allosteric regulator of metabolic enzyme function. Cell.

[bb0240] Topham D.J., Castrucci M.R., Wingo F.S., Belz G.T., Doherty P.C. (2001). The role of antigen in the localization of naive, acutely activated, and memory CD8(+) T cells to the lung during influenza pneumonia. J. Immunol..

[bb0245] Tovey M.G., Lallemand C., Thyphronitis G. (2008). Adjuvant activity of type I interferons. Biol. Chem..

[bb0250] Van Weert A.W., Geuze H.J., Groothuis B., Stoorvogel W. (2000). Primaquine interferes with membrane recycling from endosomes to the plasma membrane through a direct interaction with endosomes which does not involve neutralisation of endosomal pH nor osmotic swelling of endosomes. Eur. J. Cell Biol..

[bb0255] Wang Z., Celis E. (2015). STING activator c-di-GMP enhances the anti-tumor effects of peptide vaccines in melanoma-bearing mice. Cancer Immunol. Immunother..

[bb0260] Wang W., Golding B. (2005). The cytotoxic T lymphocyte response against a protein antigen does not decrease the antibody response to that antigen although antigen-pulsed B cells can be targets. Immunol. Lett..

[bb0265] Wang W., Merchlinsky M., Inman J., Golding B. (2005). Identification of a novel immunodominant cytotoxic T lymphocyte epitope derived from human factor VIII in a murine model of hemophilia A. Thromb. Res..

[bb0270] Xu H.C., Grusdat M., Pandyra A.A., Polz R., Huang J., Sharma P., Deenen R., Kohrer K., Rahbar R., Diefenbach A., Gibbert K., Lohning M., Hocker L., Waibler Z., Haussinger D., Mak T.W., Ohashi P.S., Lang K.S., Lang P.A. (2014). Type I interferon protects antiviral CD8 + T cells from NK cell cytotoxicity. Immunity.

[bb0275] Yin Q., Tian Y., Kabaleeswaran V., Jiang X., Tu D., Eck M.J., Chen Z.J., Wu H. (2012). Cyclic di-GMP sensing via the innate immune signaling protein STING. Mol. Cell.

[bb0280] Zehner M., Burgdorf S. (2015). Sec61 in antigen cross-presentation. Oncotarget.

[bb0285] Zehner M., Marschall A.L., Bos E., Schloetel J.G., Kreer C., Fehrenschild D., Limmer A., Ossendorp F., Lang T., Koster A.J., Dubel S., Burgdorf S. (2015). The translocon protein Sec61 mediates antigen transport from endosomes in the cytosol for cross-presentation to CD8(+) T cells. Immunity.

