# Supplementary material for: Type I IFN and not TNF, is Essential for Cyclic Di-nucleotide-elicited CTL by a Cytosolic Cross-presentation Pathway
Source: eBioMedicine. 2017 Jul 19;22:100–11. doi: 10.1016/j.ebiom.2017.07.016 (PMC5552247; doi:10.1016/j.ebiom.2017.07.016)
Supplement: Supplementary file 1 — Supplementary material [file mmc1.docx]

**Supplemental Materials**

**
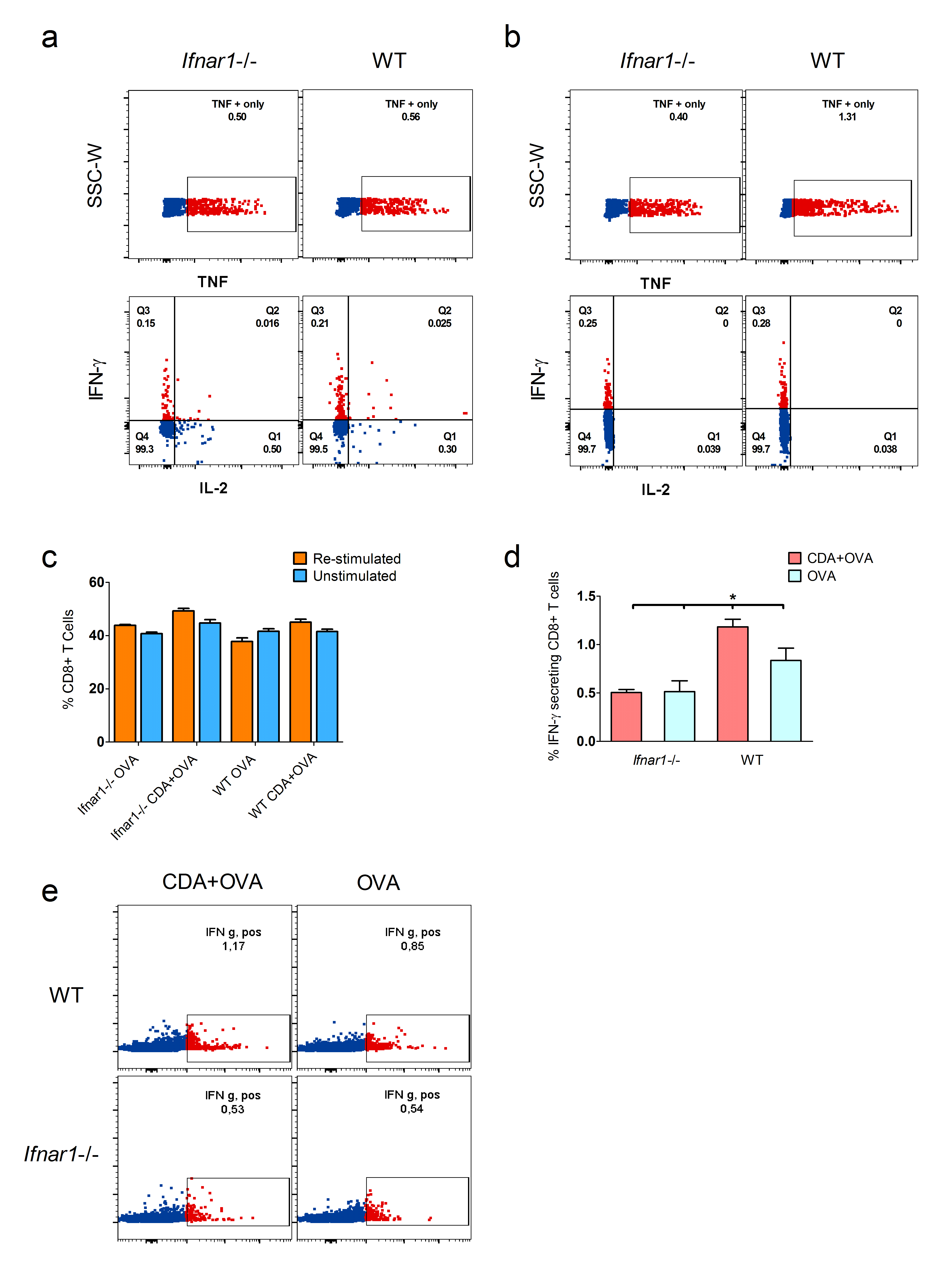
**

**Supplementary Figure 1**

**Flow cytometry dot plots for representative samples and IFN-γ secretion by CD8^+^ T cells.**

Evaluation of cytokine production (CD4^+^ and CD8^+^ T cells) and IFN-γ secretion (CD8^+^ T cells) after homologous prime and boost vaccination with CDA+OVA or OVA alone. Mice were vaccinated according to the scheme displayed on Fig 1a. Representative flow cytometry dot plots of intracellular cytokine stainings in T cells from *Ifnar1*-/- and WT mice. CD4^+^ **a)** or CD8^+^ T cell **b)** were stained with antibodies against TNF, IL-2 and IFN-γ after reactivation with OVA, secretion inhibition by brefeldin/monensin and cell permeabilization. Cytokine staining for TNF (upper panels) and IFN-γ vs IL-2 (lower panels) are plotted in **a)** and **b)**. **c)** Percentage of CD8^+^ T cells alive after *in vitro* cultivation of splenocytes from vaccinated WT and *Ifnar1*-/- mice. Cultures with (orange bars) or without (light blue bars) OVA re- stimulation are shown. **d)** Evaluation of IFN-γ secretion by CD8^+^ T cells. Cell specific IFN-γ secretion was measured following re-stimulation of splenocytes isolated from immunized mice. The results are expressed as percentage of positive cells (one representative experiment out of three), the SEM is indicated by vertical lines. Differences were considered statistically significant at p<0.05 (*) by one tailed *t* test. **e)** Representative flow cytometry dot plots from 1 of 3 independent IFN-γ secretion experiments.

**
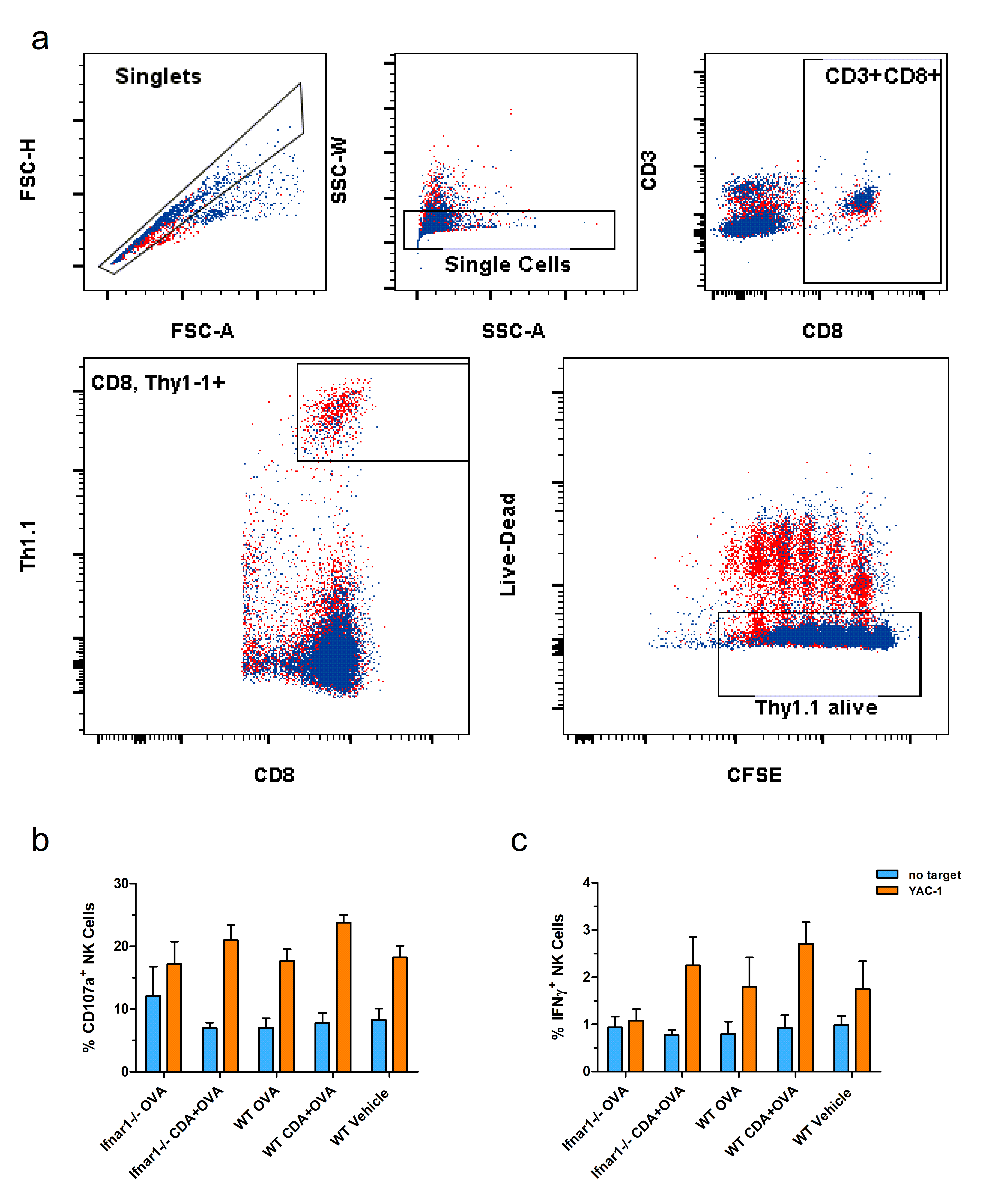
Supplementary Figure 2**

**Gating strategy for OT-I proliferation analysis and evaluation of NK activity in WT and *Ifnar1*-/- mice.**

**a)** Gating strategy used to measure CD8^+^ OT-I proliferation: after singlet gating, CD3^+^CD8^+^ cells were gated. In order to discriminate transferred CD8^+^ T cells among this population, the Thy1.1^+^ CD8^+^ cells were gated and its distribution on CFSE vs live/dead staining is plotted in the final gate used for measuring proliferation. **b**) The degranulation capacity and **c**) production of IFN-γ by NK cells isolated from WT and *Ifnar1*-/- mice was assessed after 6 h of co-incubation with NK cell target cell line YAC-1. One representative experiment out of two independent experiments is shown. SEM is indicated by vertical lines.


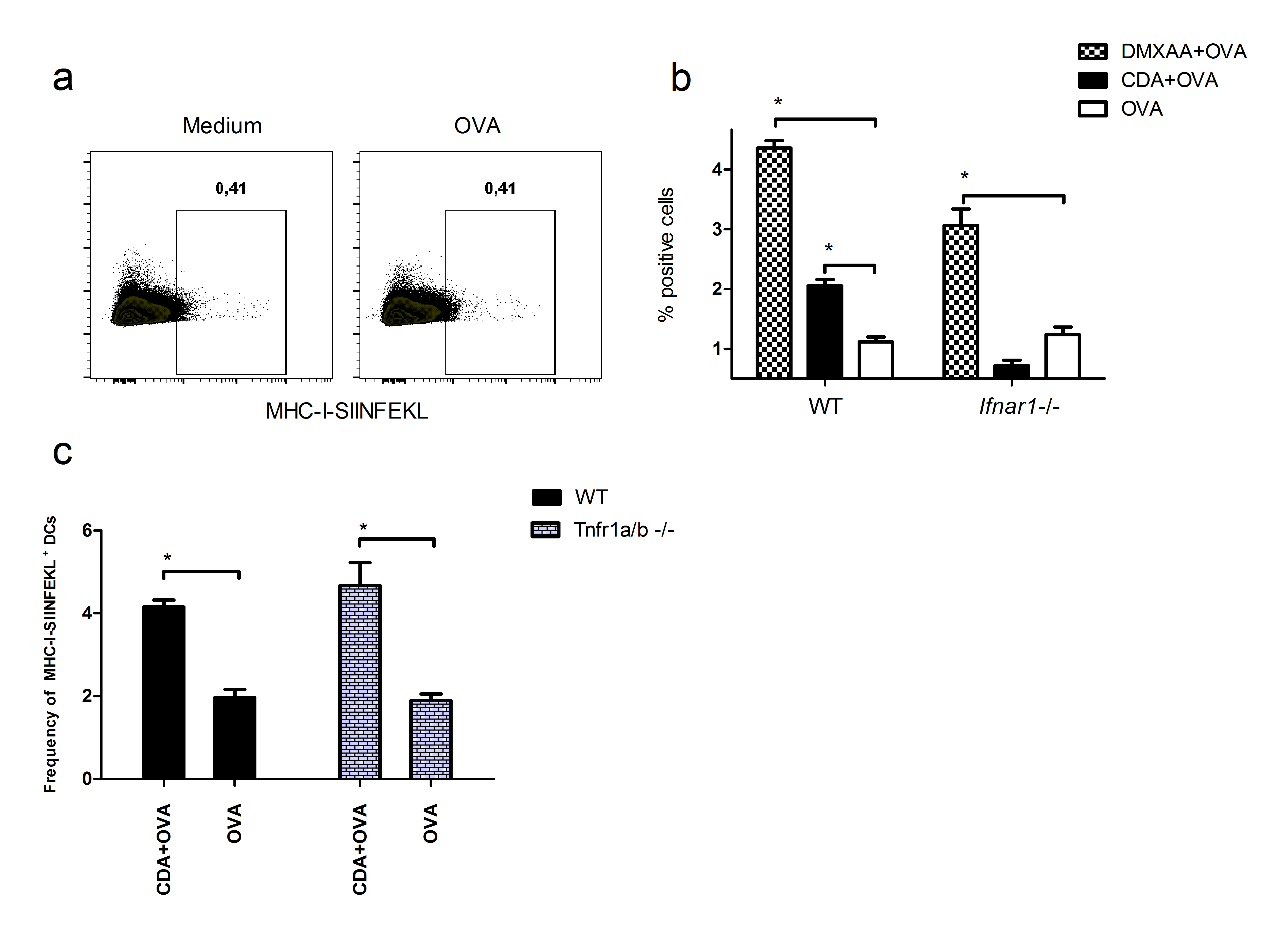


**Supplementary Figure 3**

**Type I IFN is required for CDN cross-presentation but not for DMXAA and TNF is dispensable for CDN-mediated cross-presentation.**

**a)** Raw flow cytometry graph showing the *in vivo* cross-presentation levels of vehicle control (Medium) and antigen (OVA) treatments. CD11c^+^ cells were gated from total alive splenocytes, stained with Ab 25-D1.16 (MHC-I–SIINFEKL PE positive signal). **b)** The adjuvant DMXXA, was used as Sting ligand control to check if the cross-presentation pathway elicited by CDA is shared by other Sting activators. BMDC cells were treated with the indicated combinations of antigen (OVA) and adjuvants, or vehicle. Gated CD11c^+^ cells were stained with Ab 25-D1.16 for cross-presentation. **c**) MHC-I–SIINFEKL cross-presentation analyzed by flow cytometry on CD11c^+^ gated BMDC from WT and Tnfr1a/b -/- mice. Increased percentages of cross-presentation after 24 h of treatment were detected by the antibody 25-D1.16 (MHC-I–SIINFEKL PE positive signal). Results are statistically significant (*) at *p*≤0.05 by one tailed Student *t* test. One representative out of three independent experiments is shown.





**Supplementary Figure 4**

**TAP activity in CDA-treated DC.**

TAP transport from cytosol to ER was measured in CD11c^+^ cells (DC) derived from human peripheral blood monocytes (PBMC). Incorporation of the fluorescent peptide NST-F into the ER was measured by flow cytometry. Results are statistically significant at *p*≤0.05 by one tailed Student *t* test in three independent experiments (different healthy donors), representative results from one donor are shown. SEM is indicated by vertical lines.


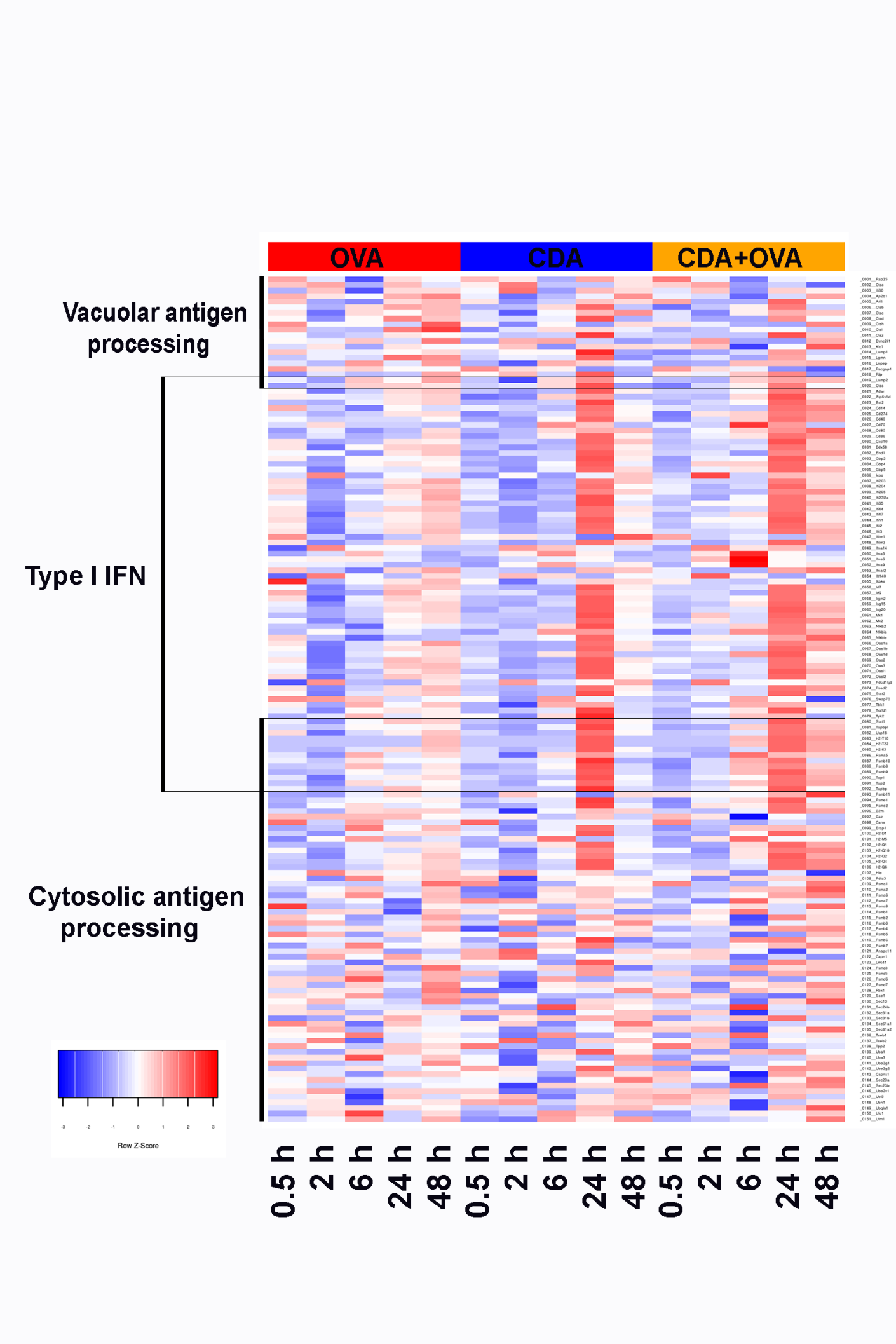


**Supplementary Figure 5**

**Microarray analysis of the effects of CDA on type I IFN related and cross-presentation genes**. The heatmap is based on logarithmic fold changes (logFCs) of genes with different expression in BDMC treated for 0.5, 2, 6, 24 and 48 h with OVA, CDA, or CDA+OVA. Z-score scaling is applied to ensure visible changes upon plotting even if genes (rows) have small logFCs. Blue and red colors indicate negative and positive values, respectively. BDMC showed selective up-regulation of numerous type I IFN-regulated genes and cytosolic antigen processing genes in cells treated for 24 h with CDA or CDA+OVA.


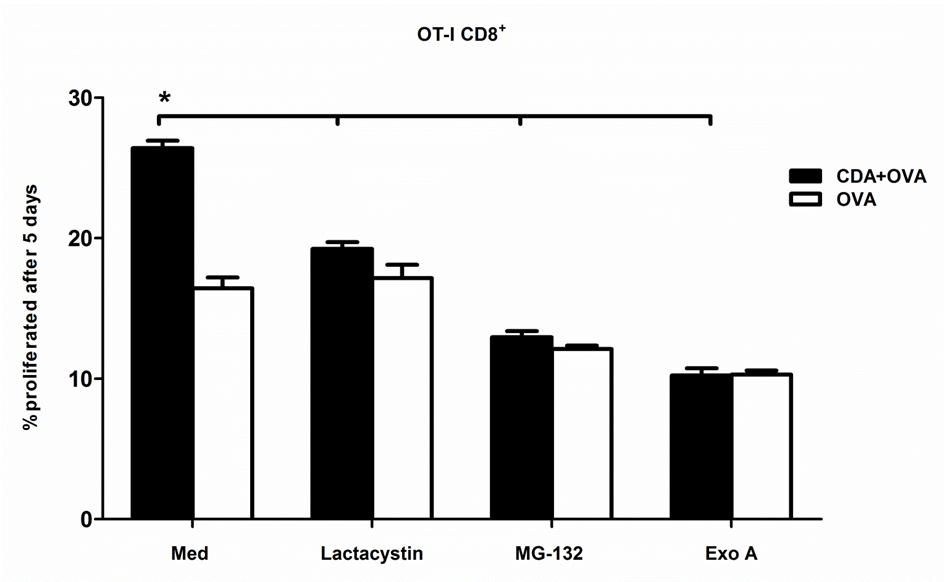


**Supplementary Figure 6**

***In vitro* blockage of the cytosolic cross-presentation pathway inhibit cross-priming of OVA-specific CD8^+^ T cells**. BMDCs treated with CDA+OVA or OVA alone in the presence or absence of cross-presentation inhibitors were co-cultivated with CFSE-labeled OT-I CD8^+^ T cells and their proliferative capacity was measured after 5 days of incubation*.* Differences were considered significant at p<0.05 (*) by one tailed *t* test. Results are from one representative experiment out of three. SEM is indicated by vertical lines.


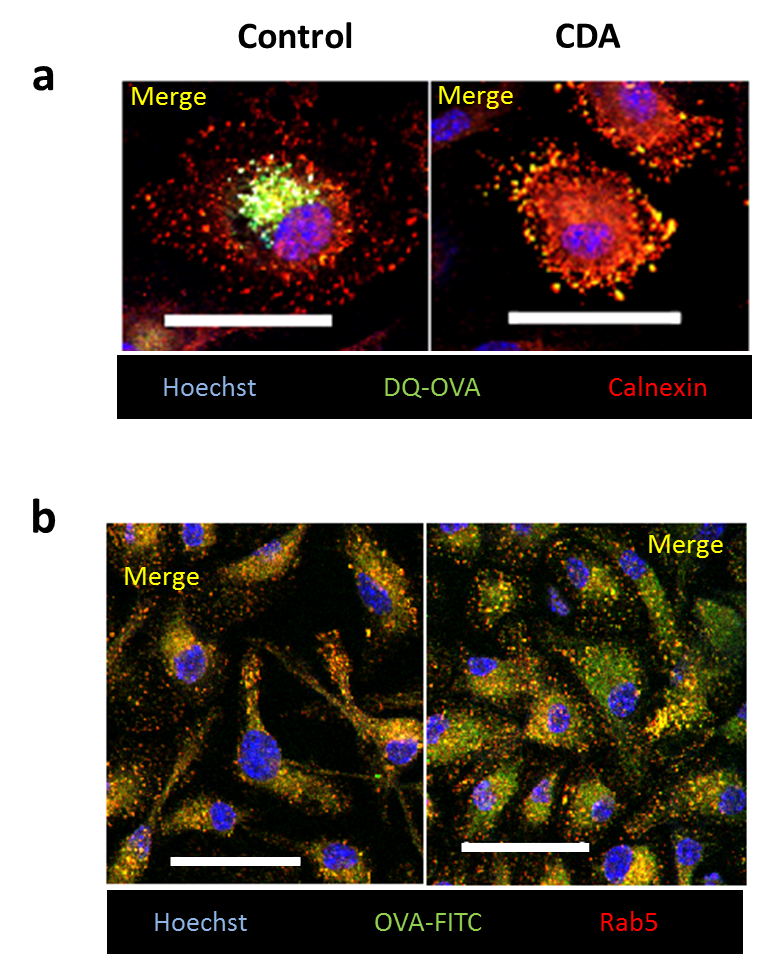


Control CDA

a

b

**Supplementary Figure 7**

**Microscopic analysis of processed antigen subcellular localization in CDA-treated BMDC.**

**a**) BMDC were incubated during 4 h in the presence of CDA+DQ-OVA or DQ-OVA alone. Antigen processing was visualized by green signal in the FITC channel and the ER marker calnexin was visualized in the red channel. **b**) BMDC were treated with OVA-FITC in the presence or absence of CDA for 15 min, washed and then further incubated for 4 h under the same conditions but without labeled antigen. Antigen processing was visualized by green signal in the FITC channel and the early endosome marker Rab5 was visualized in the red channel. Nuclear staining was performed with Hoechst 33258. Bars = 25 µm.

**Supplementary Table 1**

**Logarithmic fold changes (log FCs) of genes at 24 h.**

Selected genes from the groups considered in the heatmap (supplementary Fig 4) are given in the same order for reference (first column).
A blue bar in the column *Data set* indicate genes involved in vacuolar antigen processing while a red bar specifies genes considered for type I IFN-regulation, and the yellow bar indicates genes involved in cytosolic antigen processing.

| **ID_Gene Symbol** | **Data set** | | **Ensembl ID** | **Gene description** | **OVA** | **CDA** | **CDA+OVA** |
| --- | --- | --- | --- | --- | --- | --- | --- |
| _0001__Rab35 |  |  | ENSMUSG00000029518 | RAB35, member RAS oncogene family | 0.022 | 0.141 | -0.085 |
| _0002__Ctse |  |  | ENSMUSG00000004552 | cathepsin E | 0.109 | -0.487 | -0.465 |
| _0003__Ifi30 |  |  | ENSMUSG00000031838 | interferon gamma inducible protein 30 | 0.160 | 0.271 | 0.209 |
| _0004__Ap2b1 |  |  | ENSMUSG00000035152 | adaptor-related protein complex 2, beta 1 subunit | 0.091 | -0.027 | -0.054 |
| _0005__Arf1 |  |  | ENSMUSG00000048076 | ADP-ribosylation factor 1 | 0.092 | 0.121 | 0.274 |
| _0006__Ctsb |  |  | ENSMUSG00000021939 | cathepsin B | 0.148 | 0.234 | 0.204 |
| _0007__Ctsc |  |  | ENSMUSG00000030560 | cathepsin C | 0.093 | 0.271 | 0.287 |
| _0008__Ctsd |  |  | ENSMUSG00000007891 | cathepsin D | 0.153 | 0.222 | 0.136 |
| _0009__Ctsh |  |  | ENSMUSG00000032359 | cathepsin H | -0.513 | -0.698 | -0.651 |
| _0010__Ctsl |  |  | ENSMUSG00000021477 | cathepsin L | 0.285 | 0.109 | 0.036 |
| _0011__Ctsz |  |  | ENSMUSG00000016256 | cathepsin Z | 0.158 | 0.538 | 0.492 |
| _0012__Dync2li1 |  |  | ENSMUSG00000024253 | dynein cytoplasmic 2 light intermediate chain 1 | -0.019 | -0.029 | -0.078 |
| _0013__Klc1 |  |  | ENSMUSG00000021288 | kinesin light chain 1 | -0.025 | 0.021 | -0.080 |
| _0014__Lamp1 |  |  | ENSMUSG00000031447 | lysosomal-associated membrane protein 1 | 0.037 | 0.257 | 0.143 |
| _0015__Lgmn |  |  | ENSMUSG00000021190 | Legumain | 0.434 | 0.490 | 0.312 |
| _0016__Lnpep |  |  | ENSMUSG00000023845 | leucyl/cystinyl aminopeptidase | 0.026 | 0.175 | 0.212 |
| _0017__Racgap1 |  |  | ENSMUSG00000023015 | Rac GTPase-activating protein 1 | -0.455 | -1.219 | -1.215 |
| _0018__Rilp |  |  | ENSMUSG00000038195 | Rab interacting lysosomal protein | -0.083 | 0.345 | 0.272 |
| _0019__Lamp2 |  |  | ENSMUSG00000016534 | lysosomal-associated membrane protein 2 | 0.150 | 0.339 | 0.312 |
| _0020__Ctss |  |  | ENSMUSG00000038642 | cathepsin S | 0.130 | 0.361 | 0.273 |
| _0021__Adar |  |  | ENSMUSG00000027951 | adenosine deaminase, RNA-specific | 0.619 | 1.248 | 1.109 |
| _0022__Atp6v1d |  |  | ENSMUSG00000021114 | ATPase, H+ transporting, lysosomal V1 subunit D | 0.235 | 0.381 | 0.543 |
| _0023__Bst2 |  |  | ENSMUSG00000046718 | bone marrow stromal cell antigen 2 | 0.948 | 2.365 | 2.083 |
| _0024__Cd14 |  |  | ENSMUSG00000051439 | CD14 antigen | 0.126 | 0.595 | 0.607 |
| _0025__Cd274 |  |  | ENSMUSG00000016496 | CD274 antigen | 0.581 | 1.082 | 1.061 |
| _0026__Cd40 |  |  | ENSMUSG00000017652 | CD40 antigen | 0.314 | 1.632 | 1.575 |
| _0027__Cd70 |  |  | ENSMUSG00000019489 | CD70 antigen | 0.043 | 0.552 | 0.762 |
| _0028__Cd80 |  |  | ENSMUSG00000075122 | CD80 antigen | -0.014 | 0.698 | 0.861 |
| _0029__Cd86 |  |  | ENSMUSG00000022901 | CD86 antigen | 0.227 | 1.678 | 1.654 |
| _0030__Cxcl10 |  |  | ENSMUSG00000034855 | chemokine (C-X-C motif) ligand 10 | 1.838 | 3.888 | 4.161 |
| _0031__Ddx58 |  |  | ENSMUSG00000040296 | DEAD (Asp-Glu-Ala-Asp) box polypeptide 58 | 1.005 | 1.707 | 1.660 |
| _0032__Ehd1 |  |  | ENSMUSG00000024772 | EH-domain containing 1 | 0.212 | 0.624 | 0.484 |
| _0033__Gbp2 |  |  | ENSMUSG00000028270 | guanylate binding protein 2 | 0.606 | 1.966 | 1.928 |
| _0034__Gbp4 |  |  | ENSMUSG00000079363 | guanylate binding protein 4 | 1.178 | 2.499 | 2.354 |
| _0035__Gbp5 |  |  | ENSMUSG00000040264 | guanylate binding protein 5 | 0.724 | 2.572 | 2.558 |
| _0036__Icos |  |  | ENSMUSG00000026009 | inducible T cell co-stimulator | 0.110 | 0.073 | 0.303 |
| _0037__Ifi203 |  |  | ENSMUSG00000039997 | interferon activated gene 203 | 0.719 | 1.370 | 1.372 |
| _0038__Ifi204 |  |  | ENSMUSG00000073489 | interferon activated gene 204 | 0.988 | 1.580 | 1.694 |
| _0039__Ifi205 |  |  | ENSMUSG00000054203 | interferon activated gene 205 | 1.911 | 3.731 | 3.737 |
| _0040__Ifi27l2a |  |  | ENSMUSG00000079017 | interferon, alpha-inducible protein 27 like 2A | 0.689 | 1.941 | 1.695 |
| _0041__Ifi35 |  |  | ENSMUSG00000010358 | interferon-induced protein 35 | 0.474 | 1.673 | 1.451 |
| _0042__Ifi44 |  |  | ENSMUSG00000028037 | interferon-induced protein 44 | 2.055 | 3.729 | 3.665 |
| _0043__Ifi47 |  |  | ENSMUSG00000078920 | interferon gamma inducible protein 47 | 0.870 | 1.883 | 1.965 |
| _0044__Ifih1 |  |  | ENSMUSG00000026896 | interferon induced with helicase C domain 1 | 0.888 | 2.013 | 2.006 |
| _0045__Ifit2 |  |  | ENSMUSG00000045932 | interferon-induced protein with tetratricopeptide repeats 2 | 1.953 | 3.818 | 3.828 |
| _0046__Ifit3 |  |  | ENSMUSG00000074896 | interferon-induced protein with tetratricopeptide repeats 3 | 1.891 | 3.265 | 3.340 |
| _0047__Ifitm1 |  |  | ENSMUSG00000025491 | interferon induced transmembrane protein 1 | -0.221 | -0.454 | -0.252 |
| _0048__Ifitm3 |  |  | ENSMUSG00000025492 | interferon induced transmembrane protein 3 | 0.588 | 1.014 | 1.084 |
| _0049__Ifna14 |  |  | ENSMUSG00000095896 | interferon, alpha 14 | 0.144 | 0.116 | 0.284 |
| _0050__Ifna5 |  |  | ENSMUSG00000096682 | interferon alpha 5 | 0.146 | 0.278 | 0.239 |
| _0051__Ifna6 |  |  | ENSMUSG00000096059 | interferon alpha 6 | 0.206 | 0.283 | 0.302 |
| _0052__Ifna9 |  |  | ENSMUSG00000095270 | interferon alpha 9 | -0.004 | 0.069 | 0.132 |
| _0053__Ifnar2 |  |  | ENSMUSG00000022971 | interferon (alpha and beta) receptor 2 | 0.236 | 0.293 | 0.336 |
| _0054__Ift140 |  |  | ENSMUSG00000024169 | intraflagellar transport 140 | -0.151 | -0.183 | -0.194 |
| _0055__Ikbke |  |  | ENSMUSG00000042349 | inhibitor of kappaB kinase epsilon | 0.208 | 0.319 | 0.171 |
| _0056__Irf7 |  |  | ENSMUSG00000025498 | interferon regulatory factor 7 | 1.965 | 3.220 | 3.021 |
| _0057__Irf9 |  |  | ENSMUSG00000002325 | interferon regulatory factor 9 | 0.647 | 0.907 | 0.928 |
| _0058__Irgm2 |  |  | ENSMUSG00000069874 | immunity-related GTPase family M member 2 | 1.141 | 2.144 | 2.005 |
| _0059__Isg15 |  |  | ENSMUSG00000035692 | ISG15 ubiquitin-like modifier | 1.463 | 3.381 | 3.197 |
| _0060__Isg20 |  |  | ENSMUSG00000039236 | interferon-stimulated protein | 1.558 | 3.095 | 3.122 |
| _0061__Mx1 |  |  | ENSMUSG00000000386 | myxovirus (influenza virus) resistance 1 | 0.839 | 2.310 | 2.169 |
| _0062__Mx2 |  |  | ENSMUSG00000023341 | myxovirus (influenza virus) resistance 2 | 1.536 | 3.433 | 3.331 |
| _0063__Nfkb2 |  |  | ENSMUSG00000025225 | nuclear factor of kappa light polypeptide gene enhancer in B cells 2, p49/p100 | 0.093 | 0.459 | 0.368 |
| _0064__Nfkbia |  |  | ENSMUSG00000021025 | nuclear factor of kappa light polypeptide gene enhancer in B cells inhibitor, alpha | 0.121 | 0.341 | 0.400 |
| _0065__Nfkbie |  |  | ENSMUSG00000023947 | nuclear factor of kappa light polypeptide gene enhancer in B cells inhibitor, epsilon | 0.043 | 0.587 | 0.450 |
| _0066__Oas1a |  |  | ENSMUSG00000052776 | 2'-5' oligoadenylate synthetase 1A | 0.883 | 1.854 | 1.723 |
| _0067__Oas1b |  |  | ENSMUSG00000029605 | 2'-5' oligoadenylate synthetase 1B | 1.333 | 2.551 | 2.542 |
| _0068__Oas1d |  |  | ENSMUSG00000032623 | 2'-5' oligoadenylate synthetase 1D | 0.157 | 0.396 | 0.468 |
| _0069__Oas2 |  |  | ENSMUSG00000032690 | 2'-5' oligoadenylate synthetase 2 | 1.375 | 2.318 | 2.123 |
| _0070__Oas3 |  |  | ENSMUSG00000032661 | 2'-5' oligoadenylate synthetase 3 | 1.554 | 2.778 | 2.525 |
| _0071__Oasl1 |  |  | ENSMUSG00000041827 | 2'-5' oligoadenylate synthetase-like 1 | 1.814 | 3.939 | 3.949 |
| _0072__Oasl2 |  |  | ENSMUSG00000029561 | 2'-5' oligoadenylate synthetase-like 2 | 1.324 | 2.168 | 2.128 |
| _0073__Pdcd1lg2 |  |  | ENSMUSG00000016498 | programmed cell death 1 ligand 2 | -0.301 | 0.018 | 0.042 |
| _0074__Rsad2 |  |  | ENSMUSG00000020641 | radical S-adenosyl methionine domain containing 2 | 1.981 | 3.713 | 3.784 |
| _0075__Stat2 |  |  | ENSMUSG00000040033 | signal transducer and activator of transcription 2 | 1.086 | 2.276 | 2.107 |
| _0076__Swap70 |  |  | ENSMUSG00000031015 | SWA-70 protein | -0.071 | -0.040 | -0.008 |
| _0077__Tbk1 |  |  | ENSMUSG00000020115 | TANK-binding kinase 1 | 0.170 | 0.555 | 0.543 |
| _0078__Trafd1 |  |  | ENSMUSG00000042726 | TRAF type zinc finger domain containing 1 | 0.516 | 1.169 | 1.072 |
| _0079__Tyk2 |  |  | ENSMUSG00000032175 | tyrosine kinase 2 | 0.060 | 0.552 | 0.258 |
| _0080__Stat1 |  |  | ENSMUSG00000026104 | signal transducer and activator of transcription 1 | 0.824 | 1.513 | 1.503 |
| _0081__Tapbpl |  |  | ENSMUSG00000038213 | TAP binding protein-like | 0.398 | 1.310 | 1.139 |
| _0082__Usp18 |  |  | ENSMUSG00000030107 | ubiquitin specific peptidase 18 | 1.144 | 2.487 | 2.479 |
| _0085__H2-K1 |  |  | ENSMUSG00000061232 | histocompatibility 2, K1, K region | 0.390 | 0.829 | 0.804 |
| _0086__Psma5 |  |  | ENSMUSG00000068749 | proteasome (prosome, macropain) subunit, alpha type 5 | 0.122 | 0.559 | 0.438 |
| _0087__Psmb10 |  |  | ENSMUSG00000031897 | proteasome (prosome, macropain) subunit, beta type 10 | 0.346 | 1.452 | 1.105 |
| _0088__Psmb8 |  |  | ENSMUSG00000024338 | proteasome (prosome, macropain) subunit, beta type 8 (large multifunctional peptidase 7) | 0.082 | 1.004 | 0.816 |
| _0089__Psmb9 |  |  | ENSMUSG00000096727 | proteasome (prosome, macropain) subunit, beta type 9 (large multifunctional peptidase 2) | 0.324 | 1.204 | 1.209 |
| _0090__Tap1 |  |  | ENSMUSG00000037321 | transporter 1, ATP-binding cassette, sub-family B (MDR/TAP) | 0.613 | 1.434 | 1.290 |
| _0091__Tap2 |  |  | ENSMUSG00000024339 | transporter 2, ATP-binding cassette, sub-family B (MDR/TAP) | 0.292 | 1.142 | 1.083 |
| _0092__Tapbp |  |  | ENSMUSG00000024308 | TAP binding protein | 0.559 | 1.087 | 0.925 |
| _0093__Psmb11 |  |  | ENSMUSG00000072423 | proteasome (prosome, macropain) subunit, beta type, 11 | 0.103 | -0.090 | 0.156 |
| _0094__Psme1 |  |  | ENSMUSG00000022216 | proteasome (prosome, macropain) 28 subunit, alpha | 0.266 | 0.921 | 0.814 |
| _0095__Psme2 |  |  | ENSMUSG00000079197 | proteasome (prosome, macropain) 28 subunit, beta | 0.119 | 0.893 | 0.728 |
| _0096__B2m |  |  | ENSMUSG00000060802 | beta-2 microglobulin | 0.159 | 0.190 | 0.318 |
| _0097__Calr |  |  | ENSMUSG00000003814 | Calreticulin | -0.037 | -0.048 | -0.102 |
| _0098__Canx |  |  | ENSMUSG00000020368 | Calnexin | -0.640 | -0.515 | -0.779 |
| _0099__Erap1 |  |  | ENSMUSG00000021583 | endoplasmic reticulum aminopeptidase 1 | 0.143 | 0.502 | 0.313 |
| _0100__H2-D1 |  |  | ENSMUSG00000073411 | histocompatibility 2, D region locus 1 | 0.242 | 0.575 | 0.533 |
| _0101__H2-M5 |  |  | ENSMUSG00000024459 | histocompatibility 2, M region locus 5 | -0.037 | -0.066 | 0.044 |
| _0102__H2-Q1 |  |  | ENSMUSG00000079507 | histocompatibility 2, Q region locus 1 | 0.231 | 0.580 | 0.584 |
| _0103__H2-Q10 |  |  | ENSMUSG00000067235 | histocompatibility 2, Q region locus 10 | 0.108 | 0.565 | 0.592 |
| _0104__H2-Q2 |  |  | ENSMUSG00000091705 | histocompatibility 2, Q region locus 2 | 0.273 | 0.823 | 0.963 |
| _0105__H2-Q4 |  |  | ENSMUSG00000035929 | histocompatibility 2, Q region locus 4 | 0.737 | 2.014 | 1.877 |
| _0106__H2-Q6 |  |  | ENSMUSG00000073409 | histocompatibility 2, Q region locus 6 | 0.800 | 1.908 | 1.794 |
| _0107__Hfe |  |  | ENSMUSG00000006611 | hemochromatosis | -0.141 | -0.010 | -0.215 |
| _0108__Pdia3 |  |  | ENSMUSG00000027248 | protein disulfide isomerase associated 3 | -0.017 | -0.040 | 0.031 |
| _0109__Psma1 |  |  | ENSMUSG00000030751 | proteasome (prosome, macropain) subunit, alpha type 1 | -0.637 | -0.405 | -0.548 |
| _0110__Psma2 |  |  | ENSMUSG00000015671 | proteasome (prosome, macropain) subunit, alpha type 2 | -0.059 | 0.220 | 0.256 |
| _0111__Psma6 |  |  | ENSMUSG00000021024 | proteasome (prosome, macropain) subunit, alpha type 6 | -0.036 | 0.215 | 0.310 |
| _0112__Psma7 |  |  | ENSMUSG00000027566 | proteasome (prosome, macropain) subunit, alpha type 7 | -0.212 | 0.032 | 0.018 |
| _0113__Psma8 |  |  | ENSMUSG00000036743 | proteasome (prosome, macropain) subunit, alpha type, 8 | -0.083 | 0.084 | -0.003 |
| _0114__Psmb1 |  |  | ENSMUSG00000014769 | proteasome (prosome, macropain) subunit, beta type 1 | -0.230 | -0.030 | -0.048 |
| _0115__Psmb2 |  |  | ENSMUSG00000028837 | proteasome (prosome, macropain) subunit, beta type 2 | 0.015 | 0.285 | 0.344 |
| _0116__Psmb3 |  |  | ENSMUSG00000069744 | proteasome (prosome, macropain) subunit, beta type 3 | -0.019 | -0.007 | -0.028 |
| _0117__Psmb4 |  |  | ENSMUSG00000005779 | proteasome (prosome, macropain) subunit, beta type 4 | 0.006 | 0.214 | 0.356 |
| _0118__Psmb5 |  |  | ENSMUSG00000022193 | proteasome (prosome, macropain) subunit, beta type 5 | 0.081 | -0.052 | -0.358 |
| _0119__Psmb6 |  |  | ENSMUSG00000018286 | proteasome (prosome, macropain) subunit, beta type 6 | 0.063 | 0.536 | 0.423 |
| _0120__Psmb7 |  |  | ENSMUSG00000026750 | proteasome (prosome, macropain) subunit, beta type 7 | 0.006 | 0.241 | 0.132 |
| _0121__Anapc11 |  |  | ENSMUSG00000025135 | anaphase promoting complex subunit 11 | -0.254 | -0.262 | -0.381 |
| _0122__Capn1 |  |  | ENSMUSG00000024942 | calpain 1 | 0.046 | -0.087 | -0.156 |
| _0123__Lrrc41 |  |  | ENSMUSG00000028703 | leucine rich repeat containing 41 | 0.216 | 0.526 | 0.418 |
| _0124__Psmc3 |  |  | ENSMUSG00000002102 | proteasome (prosome, macropain) 26S subunit, ATPase 3 | -0.121 | 0.158 | 0.068 |
| _0125__Psmc5 |  |  | ENSMUSG00000020708 | protease (prosome, macropain) 26S subunit, ATPase 5 | -0.428 | 0.704 | 0.505 |
| _0126__Psmd6 |  |  | ENSMUSG00000021737 | proteasome (prosome, macropain) 26S subunit, non-ATPase, 6 | -0.135 | -0.189 | -0.219 |
| _0127__Psmd7 |  |  | ENSMUSG00000039067 | proteasome (prosome, macropain) 26S subunit, non-ATPase, 7 | -0.093 | 0.092 | 0.116 |
| _0128__Rbx1 |  |  | ENSMUSG00000022400 | ring-box 1 | -0.052 | 0.034 | 0.091 |
| _0129__Sae1 |  |  | ENSMUSG00000052833 | SUMO1 activating enzyme subunit 1 | -0.339 | -0.323 | -0.474 |
| _0130__Sec13 |  |  | ENSMUSG00000030298 | SEC13 homolog (S. cerevisiae) | -0.012 | 0.152 | 0.166 |
| _0131__Sec24b |  |  | ENSMUSG00000001052 | Sec24 related gene family, member B (S. cerevisiae) | 0.036 | 0.283 | 0.047 |
| _0132__Sec31a |  |  | ENSMUSG00000035325 | Sec31 homolog A (S. cerevisiae) | -0.045 | -0.102 | -0.182 |
| _0133__Sec31b |  |  | ENSMUSG00000051984 | Sec31 homolog B (S. cerevisiae) | 0.201 | 0.189 | 0.316 |
| _0134__Sec61a1 |  |  | ENSMUSG00000030082 | Sec61 alpha 1 subunit (S. cerevisiae) | 0.003 | -0.089 | -0.140 |
| _0135__Sec61a2 |  |  | ENSMUSG00000025816 | Sec61, alpha subunit 2 (S. cerevisiae) | -0.005 | -0.086 | -0.002 |
| _0136__Tceb1 |  |  | ENSMUSG00000079658 | transcription elongation factor B (SIII), polypeptide 1 | -0.012 | -0.124 | 0.006 |
| _0137__Tceb2 |  |  | ENSMUSG00000055839 | transcription elongation factor B (SIII), polypeptide 2 | -0.017 | 0.188 | 0.077 |
| _0138__Tpp2 |  |  | ENSMUSG00000041763 | tripeptidyl peptidase II | 0.015 | -0.175 | -0.183 |
| _0139__Uba1 |  |  | ENSMUSG00000001924 | ubiquitin-like modifier activating enzyme 1 | 0.100 | 0.220 | 0.150 |
| _0140__Uba3 |  |  | ENSMUSG00000030061 | ubiquitin-like modifier activating enzyme 3 | -0.117 | -0.252 | -0.183 |
| _0141__Ube2g1 |  |  | ENSMUSG00000020794 | ubiquitin-conjugating enzyme E2G 1 | -0.599 | -0.511 | -0.501 |
| _0142__Ube2g2 |  |  | ENSMUSG00000009293 | ubiquitin-conjugating enzyme E2G 2 | -0.085 | 0.245 | 0.165 |
| _0143__Capns1 |  |  | ENSMUSG00000001794 | calpain, small subunit 1 | 0.073 | 0.145 | 0.149 |
| _0144__Sec23a |  |  | ENSMUSG00000020986 | SEC23A (S. cerevisiae) | -0.010 | 0.002 | 0.016 |
| _0145__Sec23b |  |  | ENSMUSG00000027429 | SEC23B (S. cerevisiae) | 0.111 | 0.220 | 0.254 |
| _0146__Ube2v1 |  |  | ENSMUSG00000078923 | ubiquitin-conjugating enzyme E2 variant 1 | 0.398 | -0.174 | 0.459 |
| _0147__Ubl5 |  |  | ENSMUSG00000084786 | ubiquitin-like 5 | 0.266 | 0.176 | 0.343 |
| _0148__Ubn1 |  |  | ENSMUSG00000039473 | ubinuclein 1 | -0.090 | -0.037 | -0.190 |
| _0149__Ubqln1 |  |  | ENSMUSG00000005312 | ubiquilin 1 | 0.009 | 0.045 | 0.035 |
| _0150__Ufc1 |  |  | ENSMUSG00000062963 | ubiquitin-fold modifier conjugating enzyme 1 | -0.130 | 0.100 | -0.025 |
| _0151__Ufm1 |  |  | ENSMUSG00000027746 | ubiquitin-fold modifier 1 | -0.184 | -0.031 | -0.062 |
